# Mendelian randomization study of thyroid function and anti-Müllerian hormone levels

**DOI:** 10.3389/fendo.2023.1188284

**Published:** 2023-07-20

**Authors:** Zhu Liang, Zijin Xu, Jianqiao Liu

**Affiliations:** ^1^ Department of Obstetrics and Gynecology, Center for Reproductive Medicine, Guangdong Provincial Key Laboratory of Major Obstetric Diseases, The Third Affiliated Hospital of Guangzhou Medical University, Guangzhou, China; ^2^ Department of Obstetrics and Gynecology, Center for Reproductive Medicine, Guangdong Provincial Clinical Research Center for Obstetrics and Gynecology, The Third Affiliated Hospital of Guangzhou Medical University, Guangzhou, China; ^3^ Department of Obstetrics and Gynecology, Center for Reproductive Medicine, Guangdong-Hong Kong-Macao Greater Bay Area Higher Bay Higher Education Joint Laboratory of Maternal-Fetal Medicine, The Third Affiliated Hospital of Guangzhou Medical University, Guangzhou, China; ^4^ Key Laboratory for Reproductive Medicine of Guangdong Province, The Third Affiliated Hospital of Guangzhou Medical University, Guangzhou, China

**Keywords:** thyroid function, hypothyroidism, hyperthyroidism, AMH, Mendelian randomization

## Abstract

**Objective:**

Although previous studies have reported an association between thyroid function and anti-Müllerian hormone (AMH) levels, which is considered a reliable marker of ovarian reserve, the causal relationship between them remains uncertain. This study aims to investigate whether thyrotropin (TSH), free thyroxine (fT4), hypo- and hyperthyroidism are causally linked to AMH levels.

**Methods:**

We obtained summary statistics from three sources: the ThyroidOmics Consortium (N = 54,288), HUNT + MGI + ThyroidOmics meta-analysis (N = 119,715), and the most recent AMH genome-wide association meta-analysis (N = 7,049). Two-sample MR analyses were conducted using instrumental variables representing TSH and fT4 levels within the normal range. Additionally, we conducted secondary analyses to explore the effects of hypo- and hyperthyroidism. Subgroup analyses for TSH were also performed.

**Results:**

MR analyses did not show any causality relationship between thyroid function and AMH levels, using normal range TSH, normal range fT4, subclinical hypothyroidism, subclinical hyperthyroidism and overt hypothyroidism as exposure, respectively. In addition, neither full range TSH nor TSH with individuals <50 years old was causally associated with AMH levels. MR sensitivity analyses guaranteed the robustness of all MR results, except for the association between fT4 and AMH in the no-*DIO1*+*DIO2* group.

**Conclusion:**

Our findings suggest that there was no causal association between genetically predicted thyroid function and AMH levels in the European population.

## 1 Introduction

Ovarian reserve, defined as the quantity and quality of remaining oocytes, is a crucial factor in assessing reproductive potential (1). Diminished ovarian reserve (DOR) specifically applies to women of reproductive age who experience lower responsiveness to ovarian stimulation or reduced fecundity compared to women of similar chronological age, despite maintaining regular menstrual cycles (1). Approximately 24% of infertility cases can be attributed to DOR, making it a significant contributing factor (2). However, the underlying causes of DOR remain largely unclear, and effective prevention strategies for DOR are currently lacking in clinical practice.

Thyroid function is vital for maintaining normal female reproductive function (3–5). Thyroid hormone receptors are widely distributed throughout the human female reproductive system, including ovarian tissue (6, 7). These hormones play a role in modulating the growth of preantral follicles induced by follicle-stimulating hormone (FSH) (8, 9). Insufficient thyroid hormones have been associated with decreased production of antral follicles, as indicated by *in vivo* and *in vitro* studies (10, 11). Conversely, excessive thyroid hormones can potentially impair preantral follicle growth by inhibiting granulosa cell aromatase activity (12, 13).

The association between thyroid function and ovarian reserve in human studies remains a topic of debate. One retrospective study found that among women of advanced reproductive age, those with thyroid stimulating hormone (TSH) levels >3.0 mIU/L had significantly lower levels of anti-Müllerian hormone (AMH) compared to those with TSH levels <3.0 mIU/L (5). In another retrospective study, women aged ≥35 years with subclinical hypothyroidism initiating assisted reproductive technology (ART) had a significantly higher rate of DOR (14). However, a cross-sectional study involving nearly 5,000 women failed to establish an association between thyroid autoimmunity and DOR (15). It is important to note that the nature of these epidemiological studies prevents the determination of causality. Nonetheless, investigating whether there is a causal relationship between thyroid dysfunction and DOR could shed light on underlying biological mechanisms and inform prevention strategies.

MR analysis has emerged as a popular approach for investigating potential causal relationships between exposures and outcomes (16–18). The objective of this study was to conduct a two-sample MR analysis to assess the causal association between thyroid function and AMH, a reliable marker of ovarian reserve (19). Thyroid function indicators considered in the primary analyses were normal-range TSH and free thyroxine (fT4) levels, while secondary analyses included subclinical hypothyroidism, subclinical hyperthyroidism, and overt hypothyroidism. Additionally, subgroup analyses for TSH explored the impact of normal-range TSH using another dataset, full range TSH levels, and full range TSH among individuals under 50 years old on AMH.

## 2 Methods

### 2.1 Study overview

A two-sample MR approach was applied to examine the causal association of thyroid function with circulating AMH levels. Because of only summary-level data were employed in this study, ethical approval was not required.

**Figure 1:**
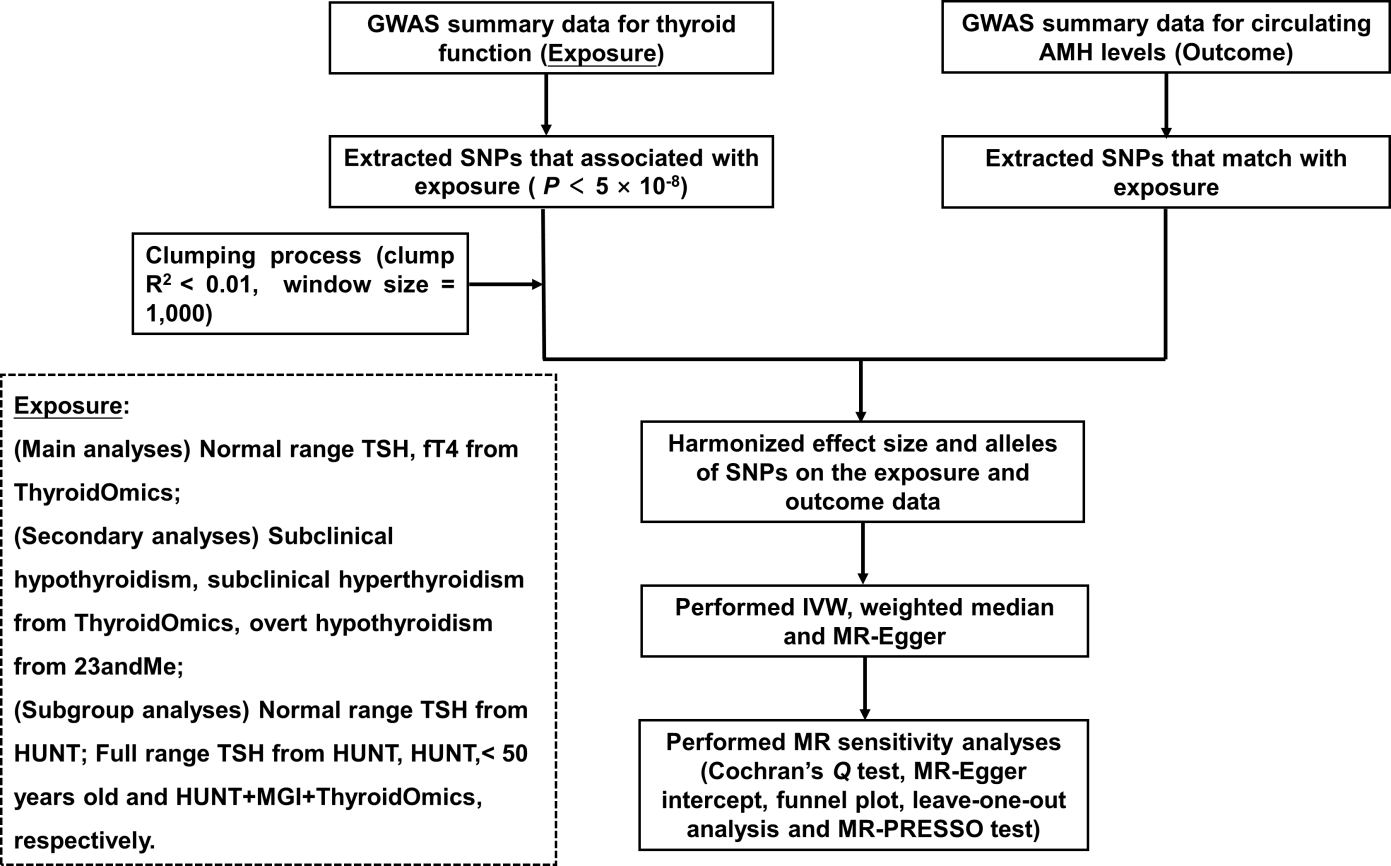
Flowchart of the Mendelian randomization study. GWAS, genome-wide association study; SNPs, single-nucleotide polymorphisms; TSH, thyroid stimulating hormone; fT4, free thyroxine; AMH, anti-Müllerian hormone; IVW, inverse-variance weighted; HUNT, a longitudinal population health study in Norway; MGI, Michigan Genomics Initiative.

The flowchart is displayed in **Figure 1**. GWAS summary data for thyroid function was the exposure, and those for circulating AMH levels served as the outcome. Main analyses investigated whether there was any causal relationship between variation in normal range thyroid function measured by TSH and fT4 and AMH levels. In order to investigate whether the potential causal effect of TSH on AMH levels were caused directly by thyroid function or indirectly by thyroid autoimmunity, single-nucleotide polymorphisms (SNPs) of the normal range TSH were categorized based on their genetically association with autoimmune thyroid disease (AITD). AITD included thyroperoxidase antibodies (TPOAb), Hashimoto’s thyroiditis and Graves’ disease. In addition, fT4 SNPs were divided according to their location within or outside Type 1 Iodothyronine Deiodinase (*DIO1*) and Type 2 Iodothyronine Deiodinase (*DIO2*) genes, which affect the bioavailability of active thyroid hormone. This was done because local tissue availability of thyroid hormones may differ from systemic plasma concentrations.

Secondary analyses examined the presence of any causal association of subclinical hypothyroidism, subclinical hyperthyroidism and overt hypothyroidism with AMH levels.

Subgroup analyses were performed to investigate the causal link between TSH and AMH levels, using the following summary statistics for (1) TSH within the normal range in the HUNT study (20) (2), full range TSH levels (HUNT study) (3), TSH levels in individuals younger (HUNT study) and (4) full range TSH levels (HUNT + MGI + ThyroidOmics meta-analysis (21)).

### 2.2 Data source

For main analyses, the exposures of interest were normal range TSH and fT4 levels, derived from a GWAS analysis by ThyroidOmics Consortium (22) (for TSH, 54,288 participants; for fT4, 49,269 participants).

For secondary analyses, thyroid function was reflected by subclinical hypothyroidism, subclinical hyperthyroidism and overt hypothyroidism. Briefly, summary statistics for subclinical hypo- and hyperthyroidism were both from ThyroidOmics Consortium (for subclinical hyperthyroidism, 1,840 cases and 49,983 controls; for subclinical hypothyroidism, 12,366 cases and 49,983 controls). Summary statistics for overt hypothyroidism were derived from 23andMe (23), with 8,000 cases and 117,000 controls.

Subgroup analyses for TSH were carried out using summary statistics for normal range TSH levels (HUNT study 53,044 participants), full range TSH levels (HUNT study, 55,342 participants), full range TSH levels in participants < 50 years old (HUNT study, 27,707 participants) and full range TSH levels (HUNT+MGI+ThyroidOmics meta-analysis (21), 119,715 participants).

Summary statistics for circulating AMH levels were retrieved from the most recent GWAS meta-analysis, which included 7049 premenopausal female European participants (24). The participants’ median age ranged from 15.3 to 48 years.

According to the detailed information of cohorts, which were recorded in the original publications of each GWAS summary statistics, all summary statistics used were derived from GWAS analyses in European ancestry, and no sample overlap was observed between thyroid function and AMH. The extensive information about the dataset included in the present study is displayed on **Table S1**.

### 2.3 Genetic variants selection criteria

SNPs were employed as instrumental variables (IVs) in this investigation. The legitimate IVs used in the MR study must fulfill three criteria: they must be strongly associated with the exposure of interest (the relevance assumption); there is no shared common cause with the outcome (the independence assumption); and IVs only impact the outcome through the path of the exposure (the exclusion restriction assumption). Therefore, only IVs meeting the following requirements were chosen for analyses: 1) genome-wide significance (*P* < 5 × 10^-8^); 2) the linkage disequilibrium (LD) of significant SNPs with r^2^ < 0.01 and clumping window size in 1,000 kb. To estimate the strength of IVs, F-statistics were calculated as F=β^2^_exposure_/SE^2^_exposure_ (25). To satisfy the first MR assumption, IVs with F-statistics less than 10 were removed for their insufficient strength. To meet the second MR assumption, IVs associated with the outcome (*P* < 5 × 10^-8^) were also removed. Subsequently, the exposure and outcome samples were harmonized to remove palindromic IVs.

### 2.4 Statistical analysis

For MR analyses, the random-effect inverse-variance weighted (IVW) approach was performed as the principal analysis, assuming all IVs to be valid and combining the effect of each IV (26). Apart from the principal analysis, the weighted median method was also conducted to examine the causality, providing a consistent causal estimate even if half of IVs suffer pleiotropy (27). In addition, MR-Egger method was also performed to estimate the causal effect, with the ability to detect and account for any directional pleiotropy (28). As part of the sensitivity analyses, MR Pleiotropy RESidual Sum and Outlier (MR-PRESSO) method also provide estimates, after detecting and correcting for horizontal pleiotropy.

Subsequently, in order to assess the validity and robustness of MR analyses, several MR sensitivity analyses were performed. Firstly, heterogeneity of the IVW model was assessed by Cochran’s Q test. Secondly, to test the third MR assumption, the presence of any directional pleiotropy was determined by MR-Egger intercept as well as funnel plot. Thirdly, the robustness of the pooled effect sizes was investigated by leave-one-out analysis. Finally, any outlier causing horizontal pleiotropy was identified and then adjusted by MR-PRESSO test.

Bonferroni correction adjusted for the number of exposures was applied, and *P* of 0.05/9 = 0.006 was defined as statistically significance. The TwoSampleMR package (version 0.5.6) in R (version 4.2.2) was used to carry out all statistical analyses in this study. The function of “run_mr_presso” (version 1.0) in the TwoSampleMR package was used to perform MR-PRESSO test.

## 3 Results

### 3.1 Genetic variants selection

The specific SNPs selected for each thyroid function phenotype can be found in **Table S2**. The F-statistics for each SNP exceeded the standard cutoff of 10, indicating a high instrument strength. The number of SNPs predicting thyroid function genetically is presented in **Tables 1**, **2**, ranging from 7 to 81 after harmonization.

**Table 1:** Results of MR analyses between liability to thyroid function and circulating AMH levels.

Exposure	N SNPs	Inverse variance weighted				MR Egger				Weighted median			
		Beta		CI	pval	Beta		CI	pval	Beta		CI	pval
Normal range TSH, ThyroidOmics	54	-0.022		-0.091- 0.047	0.531	-0.145		-0.312- 0.023	0.097	-0.031		-0.136- 0.074	0.562
AITD	15	-0.096		-0.223- 0.031	0.139	-0.232		-0.606- 0.143	0.247	-0.139		-0.315- 0.037	0.122
no-AITD	39	0.006		-0.075- 0.086	0.889	-0.117		-0.306- 0.071	0.229	-0.022		-0.147- 0.102	0.724
Normal range fT4, ThyroidOmics	23	-0.073		-0.168- 0.022	0.130	-0.061		-0.270- 0.148	0.574	-0.041		-0.182- 0.100	0.567
DIO1+DIO2	5	-0.018		-0.172- 0.136	0.819	-0.046		-0.394- 0.301	0.810	-0.013		-0.179- 0.152	0.873
no-DIO1+DIO2	18	-0.106		-0.226- 0.014	0.082	-0.130		-0.424- 0.163	0.396	-0.131		-0.307- 0.046	0.146
Subclinical hypothyroidism, ThyroidOmics	7	-0.023		-0.084- 0.039	0.471	-0.089		-0.350- 0.172	0.534	-0.030		-0.106- 0.046	0.439
Subclinical hyperthyroidism, ThyroidOmics	7	0.001		-0.046- 0.048	0.972	0.096		-0.129- 0.321	0.441	0.010		-0.052- 0.072	0.760
Overt hypothyroidism, 23andMe	13	-0.020		-0.080- 0.041	0.521	-0.063		-0.203- 0.078	0.401	-0.040		-0.124- 0.043	0.345

AMH, anti-Müllerian hormone; TSH, thyroid stimulating hormone; fT4, thyroxine (free tetraiodothyronine); GWAS, genome-wide association study.

**Table 2:** Results of MR analyses between liability to thyroid function and circulating AMH levels in the subgroup analyses for TSH.

Exposure	N SNPs	Inverse variance weighted				MR Egger				Weighted median			
		Beta		CI	pval	Beta		CI	pval	Beta		CI	pval
Normal range TSH, HUNT	38	-0.024		-0.095- 0.047	0.511	-0.093		-0.251- 0.066	0.259	-0.020		-0.115- 0.074	0.671
Full range TSH, HUNT	39	-0.025		-0.093- 0.043	0.471	-0.082		-0.232- 0.069	0.295	-0.018		-0.111- 0.075	0.707
Full range TSH, HUNT,< 50 years old	25	-0.017		-0.084- 0.050	0.616	-0.113		-0.275- 0.048	0.183	-0.025		-0.118- 0.067	0.589
Full range TSH, HUNT+MGI+ThyroidOmics	81	-0.042		-0.108- 0.024	0.215	-0.074		-0.207- 0.060	0.283	-0.063		-0.159- 0.033	0.197

AMH, anti-Müllerian hormone; TSH, thyroid stimulating hormone; fT4, thyroxine (free tetraiodothyronine); HUNT, a longitudinal population health study in Norway; MGI, Michigan Genomics Initiative; GWAS, genome-wide association study.

### 3.2 Main analyses

**Figure 2:**
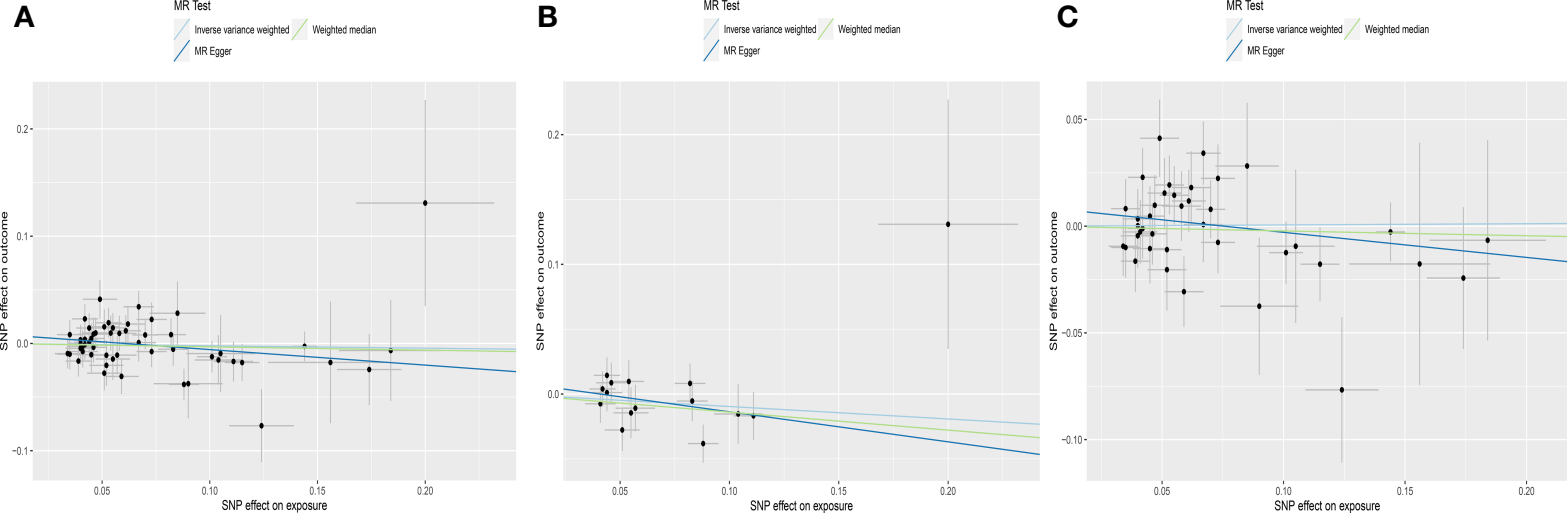
Scatter plots depicting the MR effect of normal range TSH on AMH levels in ThyroidOmics dataset. **(A)** Normal range TSH. **(B)** Normal range TSH with AITD. **(C)** Normal range TSH without AITD. The x-axis depicts the genetic link with TSH, while the y-axis depicts the genetic association with AMH levels. Each line indicates a different MR technique. AMH, anti-Müllerian hormone; TSH, thyroid stimulating hormone; AITD, autoimmune thyroid disease.

**Figure 3:**
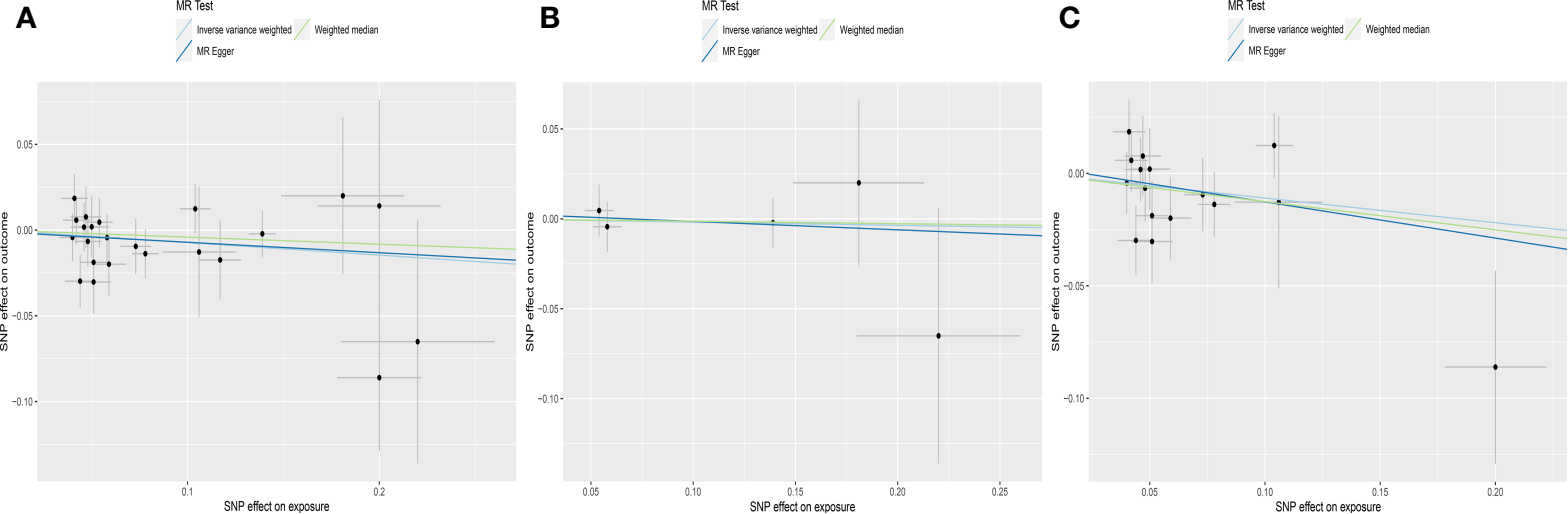
Scatter plots depicting the MR effect of normal range fT4 on AMH levels in ThyroidOmics dataset. **(A)** Normal range fT4. **(B)** Normal range fT4 with *DIO1*+*DIO2*. **(C)** Normal range fT4 without *DIO1*+*DIO2*. The x-axis depicts the genetic link with fT4, while the y-axis depicts the genetic association with AMH levels. Each line indicates a different MR technique. AMH, anti-Müllerian hormone; fT4, free thyroxine; *DIO1*, Type 1 Iodothyronine Deiodinase; *DIO2*, Type 2 Iodothyronine Deiodinase.

The results of IVW analyses showed that genetically predicted TSH and fT4 within the normal range were not causally associated with AMH levels (for TSH: OR was -0.022 at 95% CI of -0.091 to 0.047, *P* = 0.531; for fT4: OR was -0.073 at 95% CI of -0.168 to 0.022, *P* = 0.130). MR-Egger and weighted median analyses showed similar results (**Table 1**, **Figures 2**, **3**).

For subgroups analyses, MR results of TSH were robust to both AITD and no-AITD groups. Similarly, stratification by fT4 *DIO1* and *DIO2* variants did not change the results of fT4 (**Table 1**, **Figures 2**, **3**).

### 3.3 Secondary analyses

**Figure 4:**
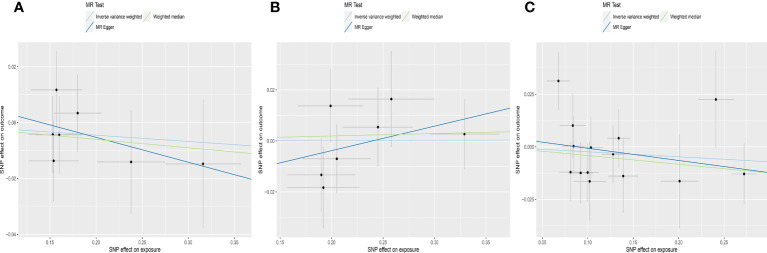
Scatter plots depicting the MR effect of thyroid function on AMH levels. **(A)** Subclinical hypothyroidism, ThyroidOmics. **(B)** Subclinical hyperthyroidism, ThyroidOmics. **(C)** Overt hypothyroidism, 23andMe. The x-axis depicts the genetic link with thyroid function, while the y-axis depicts the genetic association with AMH levels. Each line indicates a different MR technique. AMH, anti-Müllerian hormone.

IVW analyses suggested that genetic predisposition to subclinical hypothyroidism, subclinical hyperthyroidism and overt hypothyroidism were not associated with AMH levels (for subclinical hypothyroidism: OR was -0.023 at 95% CI of -0.084 to 0.039, *P* = 0.471; for subclinical hyperthyroidism: OR was 0.001 at 95% CI of -0.046 to 0.048, *P* = 0.972; for overt hypothyroidism: OR was -0.020 at 95% CI of -0.080 to 0.041, *P* = 0.521) (**Table 1**, **Figure 4**). Consistently, neither MR Egger nor weighted median indicated any causal association of subclinical hypo-, hyperthyroidism and overt hypothyroidism with AMH levels, respectively.

### 3.4 Subgroup analyses for TSH

**Figure 5:**
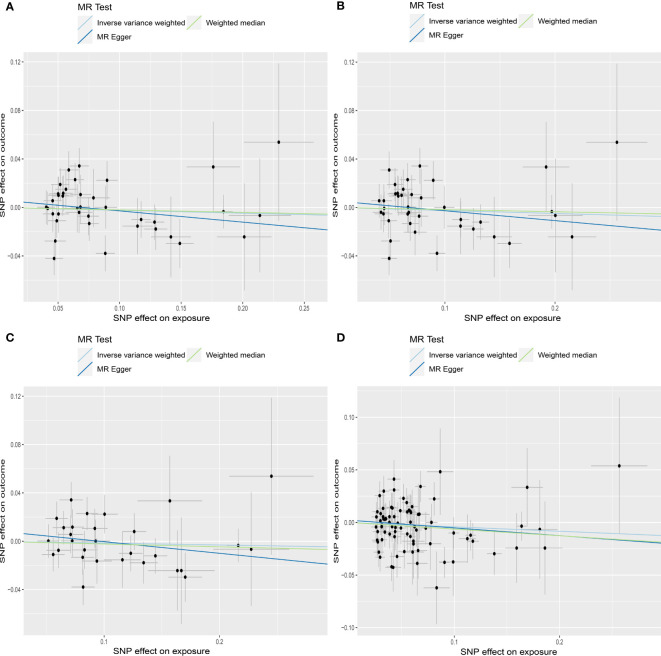
Scatter plots depicting the MR effect of each exposure on AMH levels in the subgroup analyses for TSH. **(A)** Normal range TSH, HUNT. **(B)** Full range TSH, HUNT. **(C)** Full range TSH, HUNT, < 50 years old. **(D)** Full range TSH, HUNT+MGI+ThyroidOmics. The x-axis depicts the genetic link with thyroid function, while the y-axis depicts the genetic association with AMH levels. Each line indicates a different MR technique. TSH, thyroid stimulating hormone; HUNT, a longitudinal population health study in Norway; MGI, Michigan Genomics Initiative.

MR estimates for genetically increased TSH levels did not find any significant association with AMH levels in the following groups: normal range TSH levels (HUNT study), full range TSH levels (HUNT study), full range TSH levels in participants < 50 years old (HUNT study) and full range TSH levels (HUNT+MGI+ThyroidOmics meta-analysis), respectively (**Table 2**, **Figure 5**).

### 3.5 MR sensitivity analyses

The results of Cochran’s Q test suggested that there was no heterogeneity for IVW analyses in this study (**Table S3**), indicating that was not observed in IVs for each AD. MR-Egger intercept did not find any directional pleiotropy (**Table S3**), which was also displayed in the funnel plots (**Figures S1**-**S4**). In addition, leave-one-out plots did not detect any high influence points (**Figures S5**-**S8**), with the exception of the fT4 subgroup of no-*DIO1*+*DIO2* (**Figure S6**). It suggested that the MR result for the association between fT4 and AMH was false negative in the no-*DIO1*+*DIO2* group, driven by potentially influential IVs. Thus, the outcome should be carefully interpreted, and a cautious conclusion should be reached in the fT4 subgroup of no-*DIO1*+*DIO2*. Finally, there was no outlier detected by MR-PRESSO test (**Table S3**) and the results of MR-PRESSO were shown in **Table S4**.

## 4 Discussion

This study employed a two-sample MR approach to investigate the potential causal associations between genetic predisposition to thyroid function and AMH levels. The findings indicated no conclusive evidence of a causal relationship between thyroid function liability and AMH levels. These results suggest that the associations observed in previous observational studies might be influenced by unaccounted confounding factors or shared genetic components. Several sensitivity analyses were conducted to ensure the fulfillment of the three MR assumptions in all MR results, with the exception of the fT4 and AMH association in the no-*DIO1+DIO2* group.

Numerous studies have explored the interplay between thyroid function and the female reproductive system, spanning from early follicle development to oocytes and the endometrium (4, 5, 29). The presence of thyroxin binding domains in the human ovary indicates potential direct effects of thyroid hormone on ovarian tissue (5). Women with premature ovarian insufficiency and unexplained infertility have been found to exhibit elevated TSH levels in comparison to their fertile counterparts (3). Hypothyroidism has also been associated with menstrual irregularities and anovulation (30), suggesting that thyroid dysfunction may disrupt follicular development and maturation. Consequently, it is plausible to assume that TSH levels could influence AMH levels, regardless of thyroid autoimmunity or female age. This hypothesis finds support in a study that revealed lower AMH levels in infertile women with TSH levels exceeding 2.5 mIU/L (31).

Determining causality in the previously reported association between thyroid function and AMH levels has proven challenging. This challenge stems partly from the co-occurrence of infertility and thyroid diseases, which are highly prevalent among women of reproductive age (29). The subtle manifestation of thyroid diseases, particularly subclinical hypothyroidism and hyperthyroidism, further complicates the establishment of causality. Moreover, conducting randomized controlled trials that include euthyroid individuals is impractical. To address these limitations, we adopted a MR approach as a surrogate in this study to assess the causality of the relationship between thyroid function and AMH levels.

The MR analyses conducted in this study revealed no significant association between thyroid function and AMH levels. Our findings align with a previous large-scale epidemiological study involving nearly 5,000 women, which demonstrated comparable serum levels of TSH and fT4 across different categories of ovarian reserve defined by AMH levels (15). However, caution should be exercised when interpreting our results. The absence of an association suggests that thyroid dysfunction alone does not impact ovarian reserve, potentially alleviating concerns for individuals with thyroid diseases regarding their fertility. Nonetheless, it remains important to treat thyroid diseases in order to enhance the likelihood of successful fertilization and improve embryo quality. For instance, recent guidelines on thyroid and infertility recommend initiating levothyroxine (LT4) treatment promptly in cases of overt thyroid dysfunction or when TSH levels exceed 4.0 mIU/L (32). Moreover, our study suggests that routine thyroid function testing alone may not be imperative for assessing ovarian reserve based on AMH levels, enabling informed clinical decision-making while circumventing unnecessary testing and associated costs in patients without specific indications for thyroid evaluation. Additionally, our findings underscore the necessity for further research aiming to comprehensively comprehend the relationship between thyroid function and ovarian reserve. Subsequent investigations may unveil indirect or intricate connections between these factors not captured by the present study, encouraging researchers to explore supplementary genetic, hormonal, or environmental influences on ovarian reserve based on the aforementioned findings.

Our study possesses several notable strengths. Firstly, we aimed to provide a comprehensive assessment of the association between thyroid function and AMH levels by incorporating up to nine distinct phenotypes of thyroid function. These phenotypes were derived from the most recent and extensive GWAS available. Secondly, our investigation specifically included thyroid disorders that exhibit relatively high prevalence among women of reproductive age, namely subclinical hypothyroidism, subclinical hyperthyroidism, and overt hypothyroidism (29). Thirdly, we stratified TSH levels based on their genetic association with AITD. This approach allowed us to gain insights into the separate effects of thyroid function and autoimmune factors on ovarian reserve, a distinction that is often challenging to elucidate in epidemiological studies. Fourthly, our study encompassed several subgroup analyses concerning TSH, all of which consistently yielded concordant results, thus demonstrating the robustness of our findings.

However, our study does have certain limitations that need to be acknowledged. Firstly, the inclusion of only European participants restricts the generalizability of our findings to other ancestral populations. However, this decision was made to minimize the potential effects of population stratification. Secondly, the data for AMH were obtained from the largest available genome-wide association study GWAS, but unfortunately lacked detailed demographic information (24). Consequently, stratifying AMH based on age was not achievable in our analysis. Thirdly, the summary statistics used for AMH, as the outcome variable, were based on a female-specific GWAS. However, for the exposure variables, the summary statistics were derived from a mixed-sex GWAS that included female participants. While it would have been preferable to use female-specific GWAS data for the exposures in our study, unfortunately, no such data specifically relating to thyroid function were available. To validate our findings, future research should consider conducting MR using female-specific GWAS for the exposure variables. Lastly, in this study, the assessment of genetic correlation between the exposure variables and AMH was not feasible, primarily due to the incompleteness of the summary statistics derived from prior MR studies after undergoing clumping.

## 5 Conclusion

This study does not provide evidence for a causal relationship between thyroid function and AMH levels in individuals of European descent. Instead, it suggests that the previously observed association may be influenced by environmental confounding factors.

## Data availability statement

The original contributions presented in the study are included in the article/**Supplementary Materials**, further inquiries can be directed to the corresponding author/s.

## Author contributions

ZL and JL conceived the idea for the study. ZL and ZX obtained the GWAS datasets. ZL and ZX performed the data analyses. ZL, ZX, and JL interpreted the results of the data analyses. JL was contributed to the critical revision of the manuscript. All authors read and approved the final manuscript.

## Supplementary material

Supplementary Figure 1 Funnel plots of the effect of normal range TSH on AMH levels in ThyroidOmics dataset. **(A)** Normal range TSH. **(B)** Normal range TSH with AITD. **(C)** Normal range TSH without AITD. AMH, anti-Müllerian hormone; TSH, thyroid stimulating hormone; AITD, autoimmune thyroid disease.

Supplementary Figure 2 Funnel plots of the effect of normal range fT4 on AMH levels in ThyroidOmics dataset. **(A)** Normal range fT4. **(B)** Normal range fT4 with *DIO1*+*DIO2*. **(C)** Normal range fT4 without *DIO1*+*DIO2*. AMH, anti-Müllerian hormone; fT4, free thyroxine; *DIO1*, Type 1 Iodothyronine Deiodinase; *DIO2*, Type 2 Iodothyronine Deiodinase.

Supplementary Figure 3 Funnel plots of the effect of thyroid function on AMH levels. **(A)** Subclinical hypothyroidism, ThyroidOmics. **(B)** Subclinical hyperthyroidism, ThyroidOmics. **(C)** Overt hypothyroidism, 23andMe. AMH, anti-Müllerian hormone.

Supplementary Figure 4 Funnel plots of the effect of thyroid function on AMH levels in the subgroup analyses for TSH. **A** Normal range TSH, HUNT. **B** Full range TSH, HUNT. **C** Full range TSH, HUNT, < 50 years old. **D** Full range TSH, HUNT+MGI+ThyroidOmics. TSH, thyroid stimulating hormone; HUNT, a longitudinal population health study in Norway; MGI, Michigan Genomics Initiative.

Supplementary Figure 5 Leave-one-out plots of the effect of normal range TSH on AMH levels in ThyroidOmics dataset. **(A)** Normal range TSH. **(B)** Normal range TSH with AITD. **(C)** Normal range TSH without AITD. AMH, anti-Müllerian hormone; TSH, thyroid stimulating hormone; AITD, autoimmune thyroid disease.

Supplementary Figure 6 Leave-one-out plots of the effect of normal range fT4 on AMH levels in ThyroidOmics dataset. **(A)** Normal range fT4. **(B)** Normal range fT4 with *DIO1*+*DIO2*. **(C)** Normal range fT4 without *DIO1*+*DIO2*. AMH, anti-Müllerian hormone; fT4, free thyroxine; *DIO1*, Type 1 Iodothyronine Deiodinase; *DIO2*, Type 2 Iodothyronine Deiodinase.

Supplementary Figure 7 Leave-one-out plots of the effect of thyroid function on AMH levels. **(A)** Subclinical hypothyroidism, ThyroidOmics. **(B)** Subclinical hyperthyroidism, ThyroidOmics. **(C)** Overt hypothyroidism, 23andMe. AMH, anti-Müllerian hormone.

Supplementary Figure 8 Leave-one-out plots of the effect of thyroid function on AMH levels in the subgroup analyses for TSH. **(A)** Normal range TSH, HUNT. **(B)** Full range TSH, HUNT. **(C)** Full range TSH, HUNT, < 50 years old. **(D)** Full range TSH, HUNT+MGI+ThyroidOmics.
