# Supplementary material for: Mendelian randomization study of thyroid function and anti-Müllerian hormone levels
Source: Front Endocrinol (Lausanne). 2023 Jul 20;14:1188284. doi: 10.3389/fendo.2023.1188284 (PMC10400324; doi:10.3389/fendo.2023.1188284)
Supplement: Supplementary file 2 [file Table_1.docx]

**Table S1.** Overview of genome-wide association studies included in this study.

| **Publication** | **GWAS** | **Thyroid function exposure** | **Population** |
| --- | --- | --- | --- |
| Teumer et al | ThyroidOmics* | Normal range TSH | 54,288 TSH levels were measured within a cohort-specific reference range in the absence of overt thyroid disease (thyroid surgery or medication use) and analyzed as a continuous variable following inverse normal transformation. |
|  |  | Normal range fT4 | 49,269  fT4 levels were measured within a cohort-specific reference range in the absence of overt thyroid disease (thyroid surgery or medication use) and analyzed as a continuous variable following inverse normal transformation. |
|  |  | Subclinical hypothyroidism | 3,440 cases and 49,983 controls  Cases defined on the basis of TSH level above the reference range, but without overt thyroid disease (thyroid surgery or medication use). Thus including primarily mild phenotype. |
|  |  | Subclinical hyperthyroidism | 1,840 cases and 49,983 controls. Cases defined on the basis of TSH level below the reference range, but without overt thyroid disease (thyroid surgery or medication use). Thus including primarily mild phenotype. |
| Pickrell et al | 23andMe | Overt hypothyroidism | 8,000 cases and 117,000 controls  Cases included subclinical and overt hypothyroidism, including thyroid surgery and medication use (23andMe). |
| Zhou et al | HUNT* | Normal range TSH | 53,044  TSH levels were between 0.5 mU/L and 4.5 mU/L |
|  |  | Full range TSH | 55,342 |
|  | HUNT with participants  younger than 50 years old | Full range TSH | 27,707 |
|  | HUNT+MGI*+ThyroidOmics  meta-analysis | Full range TSH | 119,715 |
| Verdiesen et al | meta-analysis | AMH | 7,049 premenopausal female participants of European ancestry. The median age of study participants ranged from 15.3 to 48 years across cohorts |

*: ThyroidOmics: The data set consists of a large meta-analysis for TSH performed by the ThyroidOmics consortium [http://www.thyroidomics.com] for up to 54,288 subjects of European ancestry23. Briefly, genotype data in 22 independent cohorts were imputed to 1000 Genomes,phase 1 version 360.

HUNT: The HUNT study is a longitudinal, repeatedly surveyed, population-based health study conducted in the county of Nord-Trøndelag, Norway, since 198416. Approximately 120,000 individuals have participated through 3 phases of HUNT.

MGI: The Michigan Genomics Initiative (MGI) is a repository of electronic health and genetic data collected from patients at Michigan Medicine during pre-surgical encounters, who have consented to linking of genetic and clinical data for research purposes.
