# Supplementary material for: Mendelian randomization study of thyroid function and anti-Müllerian hormone levels
Source: Front Endocrinol (Lausanne). 2023 Jul 20;14:1188284. doi: 10.3389/fendo.2023.1188284 (PMC10400324; doi:10.3389/fendo.2023.1188284)
Supplement: Supplementary file 3 [file Table_2.docx]

| **Table S2.** Summary-level instrument-exposure associations. | | | | | | | | | | | |
| --- | --- | --- | --- | --- | --- | --- | --- | --- | --- | --- | --- |
| **GWAS** | **Exposure** | **SNP** | **Effect allele** | **Other allele** | **Beta** | **Standard error** | **P** | **F statistic** | **AITD** | **Type of AITD** | **Nearest gene** |
| ThyroidOmics | Normal range fT4 | rs55679545 | A | G | 0.044 | 0.008 | 8.40E-09 | 30.250 |  |  |  |
| ThyroidOmics | Normal range fT4 | rs150816132 | G | A | 0.220 | 0.040 | 3.50E-08 | 30.250 |  |  | DIO2 |
| ThyroidOmics | Normal range fT4 | rs10119187 | T | C | 0.050 | 0.009 | 4.10E-09 | 30.864 |  |  |  |
| ThyroidOmics | Normal range fT4 | rs56069042 | A | G | 0.106 | 0.019 | 1.20E-08 | 31.125 |  |  |  |
| ThyroidOmics | Normal range fT4 | rs145019385 | T | C | 0.181 | 0.032 | 1.10E-08 | 31.993 |  |  | DIO1 |
| ThyroidOmics | Normal range fT4 | rs4149056 | C | T | 0.051 | 0.009 | 1.30E-08 | 32.111 |  |  |  |
| ThyroidOmics | Normal range fT4 | rs10984606 | G | T | 0.040 | 0.007 | 1.20E-09 | 32.653 |  |  |  |
| ThyroidOmics | Normal range fT4 | rs4954192 | C | T | 0.041 | 0.007 | 8.40E-09 | 34.306 |  |  |  |
| ThyroidOmics | Normal range fT4 | rs12323871 | C | T | 0.047 | 0.008 | 1.40E-08 | 34.516 |  |  |  |
| ThyroidOmics | Normal range fT4 | rs1080094 | G | A | 0.042 | 0.007 | 4.10E-10 | 36.000 |  |  |  |
| ThyroidOmics | Normal range fT4 | rs137964359 | C | T | 0.200 | 0.032 | 2.10E-10 | 39.062 |  |  |  |
| ThyroidOmics | Normal range fT4 | rs6785807 | G | A | 0.059 | 0.009 | 2.50E-10 | 42.975 |  |  |  |
| ThyroidOmics | Normal range fT4 | rs10946313 | T | C | 0.046 | 0.007 | 2.30E-11 | 43.184 |  |  |  |
| ThyroidOmics | Normal range fT4 | rs10818937 | C | T | 0.048 | 0.007 | 1.30E-11 | 47.020 |  |  |  |
| ThyroidOmics | Normal range fT4 | rs9356988 | G | A | 0.051 | 0.007 | 3.60E-12 | 53.082 |  |  |  |
| ThyroidOmics | Normal range fT4 | rs225014 | T | C | 0.054 | 0.007 | 1.80E-15 | 59.510 |  |  | DIO2 |
| ThyroidOmics | Normal range fT4 | rs954878 | G | A | 0.058 | 0.007 | 4.80E-19 | 68.653 |  |  | DIO1 |
| ThyroidOmics | Normal range fT4 | rs113107469 | T | C | 0.200 | 0.022 | 1.10E-19 | 82.645 |  |  |  |
| ThyroidOmics | Normal range fT4 | rs17185536 | T | C | 0.073 | 0.008 | 1.90E-19 | 83.266 |  |  |  |
| ThyroidOmics | Normal range fT4 | rs6854291 | A | G | 0.117 | 0.011 | 1.30E-24 | 113.132 |  |  |  |
| ThyroidOmics | Normal range fT4 | rs10739496 | T | C | 0.078 | 0.007 | 4.20E-30 | 124.163 |  |  |  |
| ThyroidOmics | Normal range fT4 | rs7860634 | A | G | 0.104 | 0.008 | 7.70E-44 | 169.000 |  |  |  |
| ThyroidOmics | Normal range fT4 | rs2235544 | A | C | 0.139 | 0.007 | 3.50E-08 | 394.306 |  |  | DIO1 |
| ThyroidOmics | Normal range TSH | rs1042673 | G | A | 0.055 | 0.006 | 3.60E-19 | 84.028 |  |  |  |
| ThyroidOmics | Normal range TSH | rs1045476 | A | G | 0.049 | 0.008 | 2.40E-09 | 37.516 |  |  |  |
| ThyroidOmics | Normal range TSH | rs1079418 | A | G | 0.101 | 0.007 | 8.20E-53 | 208.184 |  |  |  |
| ThyroidOmics | Normal range TSH | rs10814915 | T | C | 0.042 | 0.006 | 5.10E-12 | 49.000 |  |  |  |
| ThyroidOmics | Normal range TSH | rs10917469 | A | G | 0.111 | 0.009 | 4.00E-39 | 152.111 | yes | GD |  |
| ThyroidOmics | Normal range TSH | rs10957494 | G | A | 0.040 | 0.007 | 1.10E-09 | 32.653 |  |  |  |
| ThyroidOmics | Normal range TSH | rs11159482 | T | C | 0.085 | 0.013 | 6.30E-11 | 42.751 |  |  |  |
| ThyroidOmics | Normal range TSH | rs1119208 | T | C | 0.046 | 0.006 | 6.60E-13 | 58.778 |  |  |  |
| ThyroidOmics | Normal range TSH | rs11255790 | C | T | 0.041 | 0.007 | 6.80E-10 | 34.306 |  |  |  |
| ThyroidOmics | Normal range TSH | rs113974964 | C | T | 0.124 | 0.015 | 2.10E-17 | 68.338 |  |  |  |
| ThyroidOmics | Normal range TSH | rs1157994 | G | A | 0.090 | 0.016 | 5.30E-09 | 31.641 |  |  |  |
| ThyroidOmics | Normal range TSH | rs11639111 | T | C | 0.045 | 0.006 | 3.60E-13 | 56.250 |  |  |  |
| ThyroidOmics | Normal range TSH | rs11732089 | T | C | 0.115 | 0.008 | 1.70E-51 | 206.641 |  |  |  |
| ThyroidOmics | Normal range TSH | rs118039499 | A | C | 0.184 | 0.024 | 2.00E-14 | 58.778 |  |  |  |
| ThyroidOmics | Normal range TSH | rs1203944 | C | T | 0.051 | 0.007 | 2.40E-12 | 53.082 |  |  |  |
| ThyroidOmics | Normal range TSH | rs12089835 | T | C | 0.073 | 0.007 | 1.30E-28 | 108.755 |  |  |  |
| ThyroidOmics | Normal range TSH | rs12284404 | G | A | 0.067 | 0.007 | 2.50E-22 | 91.612 |  |  |  |
| ThyroidOmics | Normal range TSH | rs1265091 | T | C | 0.057 | 0.009 | 3.20E-11 | 40.111 | yes | GD |  |
| ThyroidOmics | Normal range TSH | rs12893151 | C | A | 0.062 | 0.008 | 1.00E-15 | 60.062 |  |  |  |
| ThyroidOmics | Normal range TSH | rs13015993 | A | G | 0.082 | 0.007 | 4.50E-32 | 137.224 | yes | GD |  |
| ThyroidOmics | Normal range TSH | rs13100823 | C | T | 0.041 | 0.007 | 6.80E-10 | 34.306 | yes | GD |  |
| ThyroidOmics | Normal range TSH | rs13329353 | T | C | 0.061 | 0.007 | 5.20E-21 | 75.939 |  |  |  |
| ThyroidOmics | Normal range TSH | rs139149784 | A | G | 0.156 | 0.029 | 5.00E-08 | 28.937 |  |  |  |
| ThyroidOmics | Normal range TSH | rs139424329 | G | A | 0.200 | 0.032 | 5.10E-10 | 39.063 | yes | HT |  |
| ThyroidOmics | Normal range TSH | rs1663070 | C | T | 0.046 | 0.007 | 3.50E-11 | 43.184 | yes | GD |  |
| ThyroidOmics | Normal range TSH | rs16856540 | C | T | 0.055 | 0.008 | 7.80E-11 | 47.266 | yes | GD |  |
| ThyroidOmics | Normal range TSH | rs17020122 | T | C | 0.104 | 0.011 | 5.30E-20 | 89.388 | yes | HT, TPOAb |  |
| ThyroidOmics | Normal range TSH | rs17477923 | T | C | 0.083 | 0.007 | 2.60E-33 | 140.592 | yes | TPOAb |  |
| ThyroidOmics | Normal range TSH | rs17767491 | A | G | 0.088 | 0.007 | 3.40E-42 | 158.041 | yes | TPOAb, GD |  |
| ThyroidOmics | Normal range TSH | rs2127387 | A | G | 0.144 | 0.006 | 1.10E-117 | 576.000 |  |  |  |
| ThyroidOmics | Normal range TSH | rs2254613 | G | T | 0.035 | 0.006 | 3.40E-08 | 34.028 |  |  |  |
| ThyroidOmics | Normal range TSH | rs2439301 | G | A | 0.059 | 0.008 | 8.20E-15 | 54.391 |  |  |  |
| ThyroidOmics | Normal range TSH | rs2739067 | G | A | 0.042 | 0.006 | 2.40E-11 | 49.000 |  |  |  |
| ThyroidOmics | Normal range TSH | rs28502438 | T | C | 0.034 | 0.006 | 3.70E-08 | 32.111 |  |  |  |
| ThyroidOmics | Normal range TSH | rs30227 | C | T | 0.047 | 0.006 | 7.60E-14 | 61.361 |  |  |  |
| ThyroidOmics | Normal range TSH | rs334725 | A | G | 0.174 | 0.015 | 2.40E-32 | 134.560 |  |  |  |
| ThyroidOmics | Normal range TSH | rs398745 | C | A | 0.052 | 0.006 | 4.00E-17 | 75.111 |  |  |  |
| ThyroidOmics | Normal range TSH | rs4445669 | C | T | 0.040 | 0.006 | 5.80E-11 | 44.444 |  |  |  |
| ThyroidOmics | Normal range TSH | rs4804413 | T | C | 0.053 | 0.006 | 8.60E-18 | 78.028 |  |  |  |
| ThyroidOmics | Normal range TSH | rs4933466 | A | G | 0.040 | 0.006 | 5.10E-10 | 44.444 |  |  |  |
| ThyroidOmics | Normal range TSH | rs56009477 | A | G | 0.052 | 0.008 | 3.70E-10 | 42.250 |  |  |  |
| ThyroidOmics | Normal range TSH | rs59334515 | C | T | 0.054 | 0.007 | 1.10E-13 | 59.510 | yes | GD |  |
| ThyroidOmics | Normal range TSH | rs59381142 | G | A | 0.058 | 0.008 | 1.70E-14 | 52.562 |  |  |  |
| ThyroidOmics | Normal range TSH | rs6535624 | A | G | 0.042 | 0.006 | 1.60E-11 | 49.000 | yes | TPOAb |  |
| ThyroidOmics | Normal range TSH | rs6724073 | T | C | 0.051 | 0.008 | 1.30E-10 | 40.641 | yes | TPOAb |  |
| ThyroidOmics | Normal range TSH | rs73022105 | T | C | 0.105 | 0.016 | 1.20E-11 | 43.066 |  |  |  |
| ThyroidOmics | Normal range TSH | rs7329958 | C | T | 0.044 | 0.007 | 1.10E-11 | 39.510 | yes | TPOAb |  |
| ThyroidOmics | Normal range TSH | rs7702192 | A | C | 0.070 | 0.006 | 2.60E-30 | 136.111 |  |  |  |
| ThyroidOmics | Normal range TSH | rs77819282 | A | G | 0.045 | 0.007 | 1.10E-09 | 41.327 |  |  |  |
| ThyroidOmics | Normal range TSH | rs8015085 | A | G | 0.067 | 0.008 | 2.40E-18 | 70.141 |  |  |  |
| ThyroidOmics | Normal range TSH | rs9298749 | C | A | 0.039 | 0.006 | 8.80E-10 | 42.250 |  |  |  |
| ThyroidOmics | Normal range TSH | rs9381266 | T | C | 0.073 | 0.007 | 1.80E-25 | 108.755 |  |  |  |
| ThyroidOmics | Normal range TSH | rs9497965 | T | C | 0.044 | 0.006 | 9.80E-13 | 53.778 | yes | GD |  |
| ThyroidOmics | Normal range TSH | rs963384 | T | C | 0.035 | 0.006 | 2.80E-08 | 34.028 |  |  |  |
| ThyroidOmics | Subclinical hyperthyroidism | rs66760320 | T | C | 0.192 | 0.035 | 4.400E-08 | 30.093 |  |  |  |
| ThyroidOmics | Subclinical hyperthyroidism | rs925488 | A | G | 0.190 | 0.033 | 1.100E-08 | 33.150 |  |  |  |
| ThyroidOmics | Subclinical hyperthyroidism | rs12138950 | C | A | 0.258 | 0.042 | 5.500E-10 | 37.735 |  |  |  |
| ThyroidOmics | Subclinical hyperthyroidism | rs8077245 | T | G | 0.205 | 0.033 | 5.000E-10 | 38.590 |  |  |  |
| ThyroidOmics | Subclinical hyperthyroidism | rs2983514 | G | A | 0.199 | 0.032 | 5.200E-10 | 38.673 |  |  |  |
| ThyroidOmics | Subclinical hyperthyroidism | rs17477923 | C | T | 0.245 | 0.034 | 6.100E-13 | 51.925 |  |  |  |
| ThyroidOmics | Subclinical hyperthyroidism | rs2046045 | T | G | 0.329 | 0.034 | 4.400E-22 | 93.634 |  |  |  |
| ThyroidOmics | Subclinical hypothyroidism | rs12449792 | T | C | 0.157 | 0.028 | 2.400E-05 | 31.440 |  |  |  |
| ThyroidOmics | Subclinical hypothyroidism | rs2983514 | A | G | 0.154 | 0.027 | 1.600E-05 | 32.532 |  |  |  |
| ThyroidOmics | Subclinical hypothyroidism | rs1382879 | C | T | 0.153 | 0.026 | 2.100E-06 | 34.629 |  |  |  |
| ThyroidOmics | Subclinical hypothyroidism | rs11675342 | T | C | 0.160 | 0.025 | 1.500E-07 | 40.960 |  |  |  |
| ThyroidOmics | Subclinical hypothyroidism | rs75491569 | C | T | 0.238 | 0.037 | 8.700E-08 | 41.376 |  |  |  |
| ThyroidOmics | Subclinical hypothyroidism | rs597808 | A | G | 0.180 | 0.026 | 3.500E-09 | 47.929 |  |  |  |
| ThyroidOmics | Subclinical hypothyroidism | rs78495697 | T | C | 0.316 | 0.041 | 2.600E-11 | 59.403 |  |  |  |
| HUNT+MGI+ThyroidOmics meta-analysis | Full range TSH | rs5997969 | C | T | -0.027 | 0.005 | 4.37E-08 | 30.007 |  |  |  |
| HUNT+MGI+ThyroidOmics meta-analysis | Full range TSH | rs30233 | A | G | -0.026 | 0.005 | 3.29E-08 | 30.036 |  |  |  |
| HUNT+MGI+ThyroidOmics meta-analysis | Full range TSH | rs72682433 | C | T | 0.042 | 0.008 | 4.22E-08 | 30.268 |  |  |  |
| HUNT+MGI+ThyroidOmics meta-analysis | Full range TSH | rs10878986 | C | T | -0.027 | 0.005 | 2.40E-08 | 30.458 |  |  |  |
| HUNT+MGI+ThyroidOmics meta-analysis | Full range TSH | rs74888443 | T | C | 0.060 | 0.011 | 2.70E-08 | 30.902 |  |  |  |
| HUNT+MGI+ThyroidOmics meta-analysis | Full range TSH | rs9865818 | G | A | -0.026 | 0.005 | 3.42E-08 | 30.982 |  |  |  |
| HUNT+MGI+ThyroidOmics meta-analysis | Full range TSH | rs10421676 | G | A | 0.027 | 0.005 | 1.05E-08 | 32.720 |  |  |  |
| HUNT+MGI+ThyroidOmics meta-analysis | Full range TSH | rs1373851 | T | C | -0.061 | 0.011 | 6.09E-09 | 33.473 |  |  |  |
| HUNT+MGI+ThyroidOmics meta-analysis | Full range TSH | rs4393429 | C | T | -0.030 | 0.005 | 8.75E-09 | 33.557 |  |  |  |
| HUNT+MGI+ThyroidOmics meta-analysis | Full range TSH | rs3731890 | T | C | 0.028 | 0.005 | 7.02E-09 | 33.711 |  |  |  |
| HUNT+MGI+ThyroidOmics meta-analysis | Full range TSH | rs4719486 | A | G | -0.027 | 0.005 | 8.44E-09 | 33.908 |  |  |  |
| HUNT+MGI+ThyroidOmics meta-analysis | Full range TSH | rs77994712 | G | C | -0.066 | 0.011 | 1.44E-09 | 36.563 |  |  |  |
| HUNT+MGI+ThyroidOmics meta-analysis | Full range TSH | rs6085658 | T | C | -0.029 | 0.005 | 1.32E-09 | 37.177 |  |  |  |
| HUNT+MGI+ThyroidOmics meta-analysis | Full range TSH | rs6989821 | C | T | 0.031 | 0.005 | 6.92E-10 | 38.816 |  |  |  |
| HUNT+MGI+ThyroidOmics meta-analysis | Full range TSH | rs72978712 | C | T | 0.044 | 0.007 | 9.42E-11 | 41.446 |  |  |  |
| HUNT+MGI+ThyroidOmics meta-analysis | Full range TSH | rs12029562 | A | G | -0.030 | 0.005 | 1.66E-10 | 41.677 |  |  |  |
| HUNT+MGI+ThyroidOmics meta-analysis | Full range TSH | rs12743883 | G | A | 0.031 | 0.005 | 1.00E-10 | 42.463 |  |  |  |
| HUNT+MGI+ThyroidOmics meta-analysis | Full range TSH | rs3184504 | C | T | -0.030 | 0.005 | 7.37E-11 | 42.529 |  |  |  |
| HUNT+MGI+ThyroidOmics meta-analysis | Full range TSH | rs9298749 | C | A | 0.031 | 0.005 | 5.79E-11 | 43.011 |  |  |  |
| HUNT+MGI+ThyroidOmics meta-analysis | Full range TSH | rs1801690 | G | C | -0.065 | 0.010 | 2.76E-11 | 44.187 |  |  |  |
| HUNT+MGI+ThyroidOmics meta-analysis | Full range TSH | rs10735341 | G | A | 0.046 | 0.007 | 1.76E-11 | 44.960 |  |  |  |
| HUNT+MGI+ThyroidOmics meta-analysis | Full range TSH | rs62174422 | G | T | -0.083 | 0.012 | 2.09E-11 | 45.103 |  |  |  |
| HUNT+MGI+ThyroidOmics meta-analysis | Full range TSH | rs751171 | C | T | 0.033 | 0.005 | 1.04E-11 | 45.838 |  |  |  |
| HUNT+MGI+ThyroidOmics meta-analysis | Full range TSH | rs6717283 | G | A | 0.046 | 0.007 | 1.04E-11 | 46.294 |  |  |  |
| HUNT+MGI+ThyroidOmics meta-analysis | Full range TSH | rs1045476 | G | A | -0.043 | 0.006 | 4.39E-12 | 47.520 |  |  |  |
| HUNT+MGI+ThyroidOmics meta-analysis | Full range TSH | rs3104389 | A | C | 0.062 | 0.009 | 5.84E-12 | 47.520 |  |  |  |
| HUNT+MGI+ThyroidOmics meta-analysis | Full range TSH | rs1045774 | G | A | 0.032 | 0.005 | 3.87E-12 | 47.816 |  |  |  |
| HUNT+MGI+ThyroidOmics meta-analysis | Full range TSH | rs35587648 | A | G | 0.032 | 0.005 | 4.53E-12 | 47.816 |  |  |  |
| HUNT+MGI+ThyroidOmics meta-analysis | Full range TSH | rs6721104 | C | A | 0.087 | 0.012 | 4.45E-12 | 48.074 |  |  |  |
| HUNT+MGI+ThyroidOmics meta-analysis | Full range TSH | rs11583886 | A | G | -0.035 | 0.005 | 3.83E-12 | 48.916 |  |  |  |
| HUNT+MGI+ThyroidOmics meta-analysis | Full range TSH | rs2993047 | A | G | -0.033 | 0.005 | 3.71E-12 | 49.007 |  |  |  |
| HUNT+MGI+ThyroidOmics meta-analysis | Full range TSH | rs700750 | A | C | 0.034 | 0.005 | 7.90E-13 | 50.453 |  |  |  |
| HUNT+MGI+ThyroidOmics meta-analysis | Full range TSH | rs11592436 | G | C | 0.043 | 0.006 | 7.43E-13 | 51.993 |  |  |  |
| HUNT+MGI+ThyroidOmics meta-analysis | Full range TSH | rs10415188 | G | A | -0.038 | 0.005 | 2.21E-13 | 52.886 |  |  |  |
| HUNT+MGI+ThyroidOmics meta-analysis | Full range TSH | rs4933466 | G | A | -0.035 | 0.005 | 1.37E-13 | 53.780 |  |  |  |
| HUNT+MGI+ThyroidOmics meta-analysis | Full range TSH | rs1157994 | G | A | 0.099 | 0.013 | 1.58E-13 | 54.441 |  |  |  |
| HUNT+MGI+ThyroidOmics meta-analysis | Full range TSH | rs11926459 | C | T | -0.034 | 0.005 | 1.47E-13 | 55.362 |  |  |  |
| HUNT+MGI+ThyroidOmics meta-analysis | Full range TSH | rs7966590 | A | G | 0.039 | 0.005 | 5.93E-14 | 55.554 |  |  |  |
| HUNT+MGI+ThyroidOmics meta-analysis | Full range TSH | rs34046483 | A | G | 0.077 | 0.010 | 5.74E-14 | 56.660 |  |  |  |
| HUNT+MGI+ThyroidOmics meta-analysis | Full range TSH | rs28133 | C | T | -0.066 | 0.009 | 1.25E-14 | 59.147 |  |  |  |
| HUNT+MGI+ThyroidOmics meta-analysis | Full range TSH | rs1265091 | T | C | 0.055 | 0.007 | 4.97E-15 | 60.525 |  |  |  |
| HUNT+MGI+ThyroidOmics meta-analysis | Full range TSH | rs12590163 | C | T | -0.039 | 0.005 | 1.53E-16 | 68.642 |  |  |  |
| HUNT+MGI+ThyroidOmics meta-analysis | Full range TSH | rs4445669 | C | T | 0.038 | 0.005 | 4.59E-17 | 70.251 |  |  |  |
| HUNT+MGI+ThyroidOmics meta-analysis | Full range TSH | rs7318607 | A | G | 0.041 | 0.005 | 8.09E-17 | 70.412 |  |  |  |
| HUNT+MGI+ThyroidOmics meta-analysis | Full range TSH | rs179256 | G | T | -0.067 | 0.008 | 2.82E-17 | 71.129 |  |  |  |
| HUNT+MGI+ThyroidOmics meta-analysis | Full range TSH | rs2242602 | T | A | 0.043 | 0.005 | 1.58E-17 | 73.991 |  |  |  |
| HUNT+MGI+ThyroidOmics meta-analysis | Full range TSH | rs9497965 | T | C | 0.040 | 0.005 | 9.73E-18 | 74.066 |  |  |  |
| HUNT+MGI+ThyroidOmics meta-analysis | Full range TSH | rs10186921 | T | C | 0.040 | 0.005 | 3.13E-18 | 74.805 |  |  |  |
| HUNT+MGI+ThyroidOmics meta-analysis | Full range TSH | rs544873 | A | G | 0.041 | 0.005 | 2.04E-18 | 77.797 |  |  |  |
| HUNT+MGI+ThyroidOmics meta-analysis | Full range TSH | rs6724073 | C | T | -0.053 | 0.006 | 2.48E-20 | 85.236 |  |  |  |
| HUNT+MGI+ThyroidOmics meta-analysis | Full range TSH | rs12942923 | A | G | -0.043 | 0.005 | 5.89E-21 | 88.140 |  |  |  |
| HUNT+MGI+ThyroidOmics meta-analysis | Full range TSH | rs11156905 | A | G | -0.044 | 0.005 | 3.98E-21 | 89.134 |  |  |  |
| HUNT+MGI+ThyroidOmics meta-analysis | Full range TSH | rs56009477 | G | A | -0.061 | 0.006 | 1.40E-21 | 91.517 |  |  |  |
| HUNT+MGI+ThyroidOmics meta-analysis | Full range TSH | rs117764941 | A | G | -0.257 | 0.027 | 7.76E-22 | 92.389 |  |  |  |
| HUNT+MGI+ThyroidOmics meta-analysis | Full range TSH | rs59148743 | C | T | -0.049 | 0.005 | 6.49E-22 | 94.284 |  |  |  |
| HUNT+MGI+ThyroidOmics meta-analysis | Full range TSH | rs310755 | A | G | -0.055 | 0.005 | 1.31E-25 | 109.680 |  |  |  |
| HUNT+MGI+ThyroidOmics meta-analysis | Full range TSH | rs59381142 | A | G | -0.059 | 0.006 | 8.83E-26 | 110.844 |  |  |  |
| HUNT+MGI+ThyroidOmics meta-analysis | Full range TSH | rs1042678 | A | G | 0.049 | 0.005 | 1.99E-26 | 113.583 |  |  |  |
| HUNT+MGI+ThyroidOmics meta-analysis | Full range TSH | rs118039499 | C | A | -0.181 | 0.017 | 6.30E-27 | 115.553 |  |  |  |
| HUNT+MGI+ThyroidOmics meta-analysis | Full range TSH | rs8020969 | T | C | -0.058 | 0.005 | 2.36E-27 | 117.346 |  |  |  |
| HUNT+MGI+ThyroidOmics meta-analysis | Full range TSH | rs1203949 | C | T | 0.060 | 0.005 | 2.88E-28 | 123.875 |  |  |  |
| HUNT+MGI+ThyroidOmics meta-analysis | Full range TSH | rs61938844 | A | G | 0.186 | 0.017 | 6.14E-29 | 124.878 |  |  |  |
| HUNT+MGI+ThyroidOmics meta-analysis | Full range TSH | rs10814915 | C | T | -0.052 | 0.005 | 1.33E-29 | 128.008 |  |  |  |
| HUNT+MGI+ThyroidOmics meta-analysis | Full range TSH | rs116909374 | T | C | -0.169 | 0.015 | 7.94E-30 | 128.484 |  |  |  |
| HUNT+MGI+ThyroidOmics meta-analysis | Full range TSH | rs1348005 | G | A | -0.057 | 0.005 | 1.09E-31 | 136.757 |  |  |  |
| HUNT+MGI+ThyroidOmics meta-analysis | Full range TSH | rs4804416 | G | T | 0.055 | 0.005 | 1.78E-32 | 143.820 |  |  |  |
| HUNT+MGI+ThyroidOmics meta-analysis | Full range TSH | rs925488 | A | G | 0.058 | 0.005 | 4.95E-34 | 149.219 |  |  |  |
| HUNT+MGI+ThyroidOmics meta-analysis | Full range TSH | rs10748781 | A | C | -0.058 | 0.005 | 6.20E-35 | 149.731 |  |  |  |
| HUNT+MGI+ThyroidOmics meta-analysis | Full range TSH | rs73234178 | A | G | -0.064 | 0.005 | 1.55E-34 | 152.722 |  |  |  |
| HUNT+MGI+ThyroidOmics meta-analysis | Full range TSH | rs12284404 | A | G | -0.068 | 0.005 | 2.72E-41 | 178.847 |  |  |  |
| HUNT+MGI+ThyroidOmics meta-analysis | Full range TSH | rs6575306 | G | A | 0.078 | 0.006 | 2.21E-42 | 189.596 |  |  |  |
| HUNT+MGI+ThyroidOmics meta-analysis | Full range TSH | rs17477923 | C | T | -0.073 | 0.005 | 8.30E-44 | 190.603 |  |  |  |
| HUNT+MGI+ThyroidOmics meta-analysis | Full range TSH | rs17020122 | T | C | 0.112 | 0.008 | 1.96E-43 | 191.441 |  |  |  |
| HUNT+MGI+ThyroidOmics meta-analysis | Full range TSH | rs334725 | A | G | 0.159 | 0.011 | 2.94E-50 | 222.941 |  |  |  |
| HUNT+MGI+ThyroidOmics meta-analysis | Full range TSH | rs9381266 | C | T | -0.081 | 0.005 | 5.11E-52 | 234.732 |  |  |  |
| HUNT+MGI+ThyroidOmics meta-analysis | Full range TSH | rs58722186 | T | C | -0.091 | 0.005 | 4.21E-78 | 346.270 |  |  |  |
| HUNT+MGI+ThyroidOmics meta-analysis | Full range TSH | rs1317983 | C | T | 0.099 | 0.005 | 2.37E-86 | 383.279 |  |  |  |
| HUNT+MGI+ThyroidOmics meta-analysis | Full range TSH | rs2928167 | G | A | -0.138 | 0.007 | 1.86E-85 | 387.920 |  |  |  |
| HUNT+MGI+ThyroidOmics meta-analysis | Full range TSH | rs11732089 | C | T | -0.118 | 0.006 | 6.77E-93 | 412.807 |  |  |  |
| HUNT+MGI+ThyroidOmics meta-analysis | Full range TSH | rs2983511 | C | G | -0.116 | 0.005 | 1.39E-120 | 538.833 |  |  |  |
| HUNT+MGI+ThyroidOmics meta-analysis | Full range TSH | rs1479567 | A | G | 0.164 | 0.005 | 1.00E-200 | 1231.188 |  |  |  |
| HUNT with participants younger than 50 years old | Full range TSH | rs4445669 | C | T | 0.052 | 0.009 | 4.42E-08 | 29.956 |  |  |  |
| HUNT with participants younger than 50 years old | Full range TSH | rs116909374 | T | C | -0.157 | 0.028 | 2.13E-08 | 31.373 |  |  |  |
| HUNT with participants younger than 50 years old | Full range TSH | rs7966590 | A | G | 0.056 | 0.010 | 1.50E-08 | 32.054 |  |  |  |
| HUNT with participants younger than 50 years old | Full range TSH | rs61938844 | A | G | 0.167 | 0.029 | 7.62E-09 | 33.369 |  |  |  |
| HUNT with participants younger than 50 years old | Full range TSH | rs4804416 | G | T | 0.059 | 0.010 | 8.74E-10 | 37.587 |  |  |  |
| HUNT with participants younger than 50 years old | Full range TSH | rs117764941 | A | G | -0.245 | 0.037 | 2.21E-11 | 44.778 |  |  |  |
| HUNT with participants younger than 50 years old | Full range TSH | rs1042678 | A | G | 0.065 | 0.010 | 9.14E-12 | 46.505 |  |  |  |
| HUNT with participants younger than 50 years old | Full range TSH | rs118039499 | C | A | -0.227 | 0.033 | 5.12E-12 | 47.641 |  |  |  |
| HUNT with participants younger than 50 years old | Full range TSH | rs12284404 | A | G | -0.072 | 0.010 | 2.02E-12 | 49.469 |  |  |  |
| HUNT with participants younger than 50 years old | Full range TSH | rs1348005 | G | A | -0.073 | 0.010 | 6.17E-13 | 51.792 |  |  |  |
| HUNT with participants younger than 50 years old | Full range TSH | rs17020122 | T | C | 0.116 | 0.016 | 4.63E-13 | 52.354 |  |  |  |
| HUNT with participants younger than 50 years old | Full range TSH | rs10748781 | A | C | -0.072 | 0.010 | 1.10E-13 | 55.172 |  |  |  |
| HUNT with participants younger than 50 years old | Full range TSH | rs73234178 | A | G | -0.083 | 0.011 | 6.14E-14 | 56.326 |  |  |  |
| HUNT with participants younger than 50 years old | Full range TSH | rs6575306 | G | A | 0.093 | 0.012 | 4.10E-14 | 57.120 |  |  |  |
| HUNT with participants younger than 50 years old | Full range TSH | rs334725 | A | G | 0.163 | 0.021 | 1.17E-14 | 59.588 |  |  |  |
| HUNT with participants younger than 50 years old | Full range TSH | rs925488 | A | G | 0.081 | 0.010 | 5.43E-16 | 65.633 |  |  |  |
| HUNT with participants younger than 50 years old | Full range TSH | rs58722186 | T | C | -0.082 | 0.010 | 2.41E-16 | 67.231 |  |  |  |
| HUNT with participants younger than 50 years old | Full range TSH | rs8020969 | T | C | -0.092 | 0.011 | 1.54E-16 | 68.113 |  |  |  |
| HUNT with participants younger than 50 years old | Full range TSH | rs9381266 | C | T | -0.100 | 0.011 | 9.25E-19 | 78.213 |  |  |  |
| HUNT with participants younger than 50 years old | Full range TSH | rs10814915 | C | T | -0.085 | 0.010 | 3.36E-19 | 80.212 |  |  |  |
| HUNT with participants younger than 50 years old | Full range TSH | rs11732089 | C | T | -0.134 | 0.012 | 3.16E-28 | 121.378 |  |  |  |
| HUNT with participants younger than 50 years old | Full range TSH | rs2928167 | G | A | -0.170 | 0.015 | 1.12E-31 | 137.148 |  |  |  |
| HUNT with participants younger than 50 years old | Full range TSH | rs1317983 | C | T | 0.123 | 0.010 | 1.53E-33 | 145.676 |  |  |  |
| HUNT with participants younger than 50 years old | Full range TSH | rs2983511 | C | G | -0.144 | 0.010 | 2.36E-44 | 195.175 |  |  |  |
| HUNT with participants younger than 50 years old | Full range TSH | rs1479567 | A | G | 0.216 | 0.010 | 3.88E-107 | 483.406 |  |  |  |
| HUNT | Full range TSH | rs7318607 | A | G | 0.042 | 0.008 | 4.59E-08 | 29.884 |  |  |  |
| HUNT | Full range TSH | rs1045774 | G | A | 0.040 | 0.007 | 4.45E-08 | 29.944 |  |  |  |
| HUNT | Full range TSH | rs59148743 | C | T | -0.044 | 0.008 | 3.31E-08 | 30.518 |  |  |  |
| HUNT | Full range TSH | rs6724073 | C | T | -0.051 | 0.008 | 2.52E-09 | 35.523 |  |  |  |
| HUNT | Full range TSH | rs179256 | G | T | -0.078 | 0.013 | 1.49E-09 | 36.543 |  |  |  |
| HUNT | Full range TSH | rs12590163 | C | T | -0.045 | 0.007 | 7.87E-10 | 37.792 |  |  |  |
| HUNT | Full range TSH | rs12942923 | A | G | -0.045 | 0.007 | 6.28E-10 | 38.233 |  |  |  |
| HUNT | Full range TSH | rs2242602 | T | A | 0.049 | 0.008 | 4.17E-10 | 39.034 |  |  |  |
| HUNT | Full range TSH | rs3104389 | A | C | 0.067 | 0.011 | 4.15E-10 | 39.039 |  |  |  |
| HUNT | Full range TSH | rs7966590 | A | G | 0.049 | 0.007 | 6.37E-11 | 42.703 |  |  |  |
| HUNT | Full range TSH | rs59381142 | A | G | -0.058 | 0.009 | 1.88E-11 | 45.087 |  |  |  |
| HUNT | Full range TSH | rs10186921 | T | C | 0.049 | 0.007 | 6.28E-12 | 47.240 |  |  |  |
| HUNT | Full range TSH | rs56009477 | G | A | -0.073 | 0.010 | 3.58E-12 | 48.341 |  |  |  |
| HUNT | Full range TSH | rs310755 | A | G | -0.061 | 0.008 | 6.71E-13 | 51.628 |  |  |  |
| HUNT | Full range TSH | rs1203949 | C | T | 0.062 | 0.009 | 4.79E-13 | 52.290 |  |  |  |
| HUNT | Full range TSH | rs1348005 | G | A | -0.057 | 0.008 | 7.93E-14 | 55.822 |  |  |  |
| HUNT | Full range TSH | rs4804416 | G | T | 0.055 | 0.007 | 5.29E-14 | 56.619 |  |  |  |
| HUNT | Full range TSH | rs1042678 | A | G | 0.055 | 0.007 | 2.56E-14 | 58.045 |  |  |  |
| HUNT | Full range TSH | rs17477923 | C | T | -0.066 | 0.008 | 4.74E-15 | 61.367 |  |  |  |
| HUNT | Full range TSH | rs118039499 | C | A | -0.200 | 0.025 | 7.16E-16 | 65.089 |  |  |  |
| HUNT | Full range TSH | rs8020969 | T | C | -0.070 | 0.008 | 1.35E-16 | 68.377 |  |  |  |
| HUNT | Full range TSH | rs925488 | A | G | 0.068 | 0.008 | 1.85E-19 | 81.397 |  |  |  |
| HUNT | Full range TSH | rs10748781 | A | C | -0.066 | 0.007 | 1.76E-19 | 81.497 |  |  |  |
| HUNT | Full range TSH | rs116909374 | T | C | -0.192 | 0.021 | 1.50E-19 | 81.805 |  |  |  |
| HUNT | Full range TSH | rs334725 | A | G | 0.145 | 0.016 | 1.08E-19 | 82.464 |  |  |  |
| HUNT | Full range TSH | rs73234178 | A | G | -0.077 | 0.008 | 4.92E-20 | 84.012 |  |  |  |
| HUNT | Full range TSH | rs10814915 | C | T | -0.066 | 0.007 | 4.78E-20 | 84.070 |  |  |  |
| HUNT | Full range TSH | rs117764941 | A | G | -0.256 | 0.028 | 2.13E-20 | 85.667 |  |  |  |
| HUNT | Full range TSH | rs17020122 | T | C | 0.114 | 0.012 | 4.12E-21 | 88.916 |  |  |  |
| HUNT | Full range TSH | rs61938844 | A | G | 0.215 | 0.022 | 8.62E-23 | 96.570 |  |  |  |
| HUNT | Full range TSH | rs12284404 | A | G | -0.077 | 0.008 | 6.39E-24 | 101.720 |  |  |  |
| HUNT | Full range TSH | rs9381266 | C | T | -0.089 | 0.009 | 1.86E-25 | 108.725 |  |  |  |
| HUNT | Full range TSH | rs6575306 | G | A | 0.099 | 0.009 | 2.17E-27 | 117.553 |  |  |  |
| HUNT | Full range TSH | rs58722186 | T | C | -0.093 | 0.008 | 1.46E-34 | 150.347 |  |  |  |
| HUNT | Full range TSH | rs11732089 | C | T | -0.126 | 0.009 | 1.59E-42 | 186.792 |  |  |  |
| HUNT | Full range TSH | rs2928167 | G | A | -0.159 | 0.011 | 7.87E-47 | 206.524 |  |  |  |
| HUNT | Full range TSH | rs1317983 | C | T | 0.114 | 0.008 | 1.26E-49 | 219.338 |  |  |  |
| HUNT | Full range TSH | rs2983511 | C | G | -0.132 | 0.008 | 2.62E-64 | 286.687 |  |  |  |
| HUNT | Full range TSH | rs1479567 | A | G | 0.197 | 0.007 | 6.21E-155 | 703.138 |  |  |  |
| HUNT | Normal range TSH | rs6724073 | C | T | -0.048 | 0.009 | 3.16E-08 | 30.604 |  |  |  |
| HUNT | Normal range TSH | rs4445669 | C | T | 0.040 | 0.007 | 3.00E-08 | 30.708 |  |  |  |
| HUNT | Normal range TSH | rs10735341 | G | A | 0.066 | 0.012 | 2.16E-08 | 31.348 |  |  |  |
| HUNT | Normal range TSH | rs12590163 | C | T | -0.042 | 0.007 | 1.93E-08 | 31.561 |  |  |  |
| HUNT | Normal range TSH | rs59148743 | C | T | -0.046 | 0.008 | 1.85E-08 | 31.643 |  |  |  |
| HUNT | Normal range TSH | rs17477923 | C | T | -0.050 | 0.009 | 4.86E-09 | 34.245 |  |  |  |
| HUNT | Normal range TSH | rs310755 | A | G | -0.050 | 0.009 | 4.30E-09 | 34.483 |  |  |  |
| HUNT | Normal range TSH | rs179256 | G | T | -0.079 | 0.013 | 2.15E-09 | 35.833 |  |  |  |
| HUNT | Normal range TSH | rs3104389 | A | C | 0.067 | 0.011 | 8.33E-10 | 37.682 |  |  |  |
| HUNT | Normal range TSH | rs59381142 | A | G | -0.054 | 0.009 | 7.12E-10 | 37.989 |  |  |  |
| HUNT | Normal range TSH | rs12942923 | A | G | -0.046 | 0.007 | 4.66E-10 | 38.815 |  |  |  |
| HUNT | Normal range TSH | rs10186921 | T | C | 0.047 | 0.007 | 1.40E-10 | 41.165 |  |  |  |
| HUNT | Normal range TSH | rs7966590 | A | G | 0.049 | 0.008 | 1.26E-10 | 41.365 |  |  |  |
| HUNT | Normal range TSH | rs1203949 | C | T | 0.056 | 0.009 | 1.05E-10 | 41.734 |  |  |  |
| HUNT | Normal range TSH | rs1042678 | A | G | 0.050 | 0.007 | 9.02E-12 | 46.531 |  |  |  |
| HUNT | Normal range TSH | rs1348005 | G | A | -0.054 | 0.008 | 2.97E-12 | 48.709 |  |  |  |
| HUNT | Normal range TSH | rs4804416 | G | T | 0.052 | 0.007 | 2.38E-12 | 49.145 |  |  |  |
| HUNT | Normal range TSH | rs2242602 | T | A | 0.059 | 0.008 | 2.55E-13 | 53.525 |  |  |  |
| HUNT | Normal range TSH | rs8020969 | T | C | -0.068 | 0.009 | 1.79E-15 | 63.282 |  |  |  |
| HUNT | Normal range TSH | rs117764941 | A | G | -0.229 | 0.028 | 6.08E-16 | 65.410 |  |  |  |
| HUNT | Normal range TSH | rs116909374 | T | C | -0.176 | 0.022 | 4.70E-16 | 65.917 |  |  |  |
| HUNT | Normal range TSH | rs118039499 | C | A | -0.214 | 0.025 | 2.93E-17 | 71.393 |  |  |  |
| HUNT | Normal range TSH | rs12284404 | A | G | -0.067 | 0.008 | 6.42E-18 | 74.387 |  |  |  |
| HUNT | Normal range TSH | rs334725 | A | G | 0.142 | 0.016 | 3.02E-18 | 75.878 |  |  |  |
| HUNT | Normal range TSH | rs10814915 | C | T | -0.064 | 0.007 | 2.94E-18 | 75.931 |  |  |  |
| HUNT | Normal range TSH | rs73234178 | A | G | -0.075 | 0.009 | 1.62E-18 | 77.103 |  |  |  |
| HUNT | Normal range TSH | rs61938844 | A | G | 0.201 | 0.022 | 1.25E-19 | 82.173 |  |  |  |
| HUNT | Normal range TSH | rs10748781 | A | C | -0.068 | 0.007 | 8.52E-20 | 82.925 |  |  |  |
| HUNT | Normal range TSH | rs17020122 | T | C | 0.115 | 0.012 | 1.38E-20 | 86.519 |  |  |  |
| HUNT | Normal range TSH | rs6575306 | G | A | 0.089 | 0.009 | 1.45E-21 | 90.975 |  |  |  |
| HUNT | Normal range TSH | rs925488 | A | G | 0.075 | 0.008 | 1.39E-22 | 95.622 |  |  |  |
| HUNT | Normal range TSH | rs9381266 | C | T | -0.090 | 0.009 | 7.59E-25 | 105.944 |  |  |  |
| HUNT | Normal range TSH | rs58722186 | T | C | -0.088 | 0.008 | 1.67E-30 | 131.776 |  |  |  |
| HUNT | Normal range TSH | rs2928167 | G | A | -0.149 | 0.011 | 8.33E-40 | 174.344 |  |  |  |
| HUNT | Normal range TSH | rs11732089 | C | T | -0.129 | 0.009 | 2.07E-43 | 190.851 |  |  |  |
| HUNT | Normal range TSH | rs1317983 | C | T | 0.117 | 0.008 | 1.48E-50 | 223.608 |  |  |  |
| HUNT | Normal range TSH | rs2983511 | C | G | -0.128 | 0.008 | 1.60E-58 | 260.143 |  |  |  |
| HUNT | Normal range TSH | rs1479567 | A | G | 0.184 | 0.008 | 1.30E-131 | 595.910 |  |  |  |
| 23andMe | Overt hypothyroidism | rs10821973 | G | A | 0.068 | 0.013 | 4.10E-08 | 27.762 |  |  |  |
| 23andMe | Overt hypothyroidism | rs1861628 | G | A | 0.083 | 0.014 | 5.60E-09 | 33.969 |  |  |  |
| 23andMe | Overt hypothyroidism | rs2466075 | G | A | 0.081 | 0.013 | 5.90E-10 | 38.822 |  |  |  |
| 23andMe | Overt hypothyroidism | rs10748781 | C | A | 0.084 | 0.013 | 1.40E-10 | 41.241 |  |  |  |
| 23andMe | Overt hypothyroidism | rs1079418 | A | G | 0.092 | 0.014 | 9.30E-12 | 45.194 |  |  |  |
| 23andMe | Overt hypothyroidism | rs6914622 | T | G | 0.103 | 0.013 | 1.20E-14 | 60.212 |  |  |  |
| 23andMe | Overt hypothyroidism | rs3087243 | G | A | 0.100 | 0.012 | 1.40E-15 | 64.234 |  |  |  |
| 23andMe | Overt hypothyroidism | rs76342258 | G | T | 0.139 | 0.016 | 8.10E-18 | 71.836 |  |  |  |
| 23andMe | Overt hypothyroidism | rs17020055 | C | A | 0.201 | 0.021 | 2.70E-22 | 91.612 |  |  |  |
| 23andMe | Overt hypothyroidism | rs1479567 | A | G | 0.128 | 0.012 | 1.40E-23 | 105.243 |  |  |  |
| 23andMe | Overt hypothyroidism | rs10774625 | A | G | 0.134 | 0.013 | 1.40E-26 | 114.918 |  |  |  |
| 23andMe | Overt hypothyroidism | rs2476601 | A | G | 0.241 | 0.020 | 2.10E-31 | 145.202 |  |  |  |
| 23andMe | Overt hypothyroidism | rs10759927 | G | A | 0.272 | 0.014 | 9.70E-90 | 377.469 |  |  |  |

GWAS, Genome-Wide Association Study; HUNT, a longitudinal population health study in Norway; MGI, Michigan Genomics Initiative; TSH, Thyroid-Stimulating Hormone; fT4, Free Thyroxine; SNP, Single Nucleotide Polymorphism; AITD, autoimmune thyroid disease; GD, Graves' disease; HT, Hashimoto's thyroiditis; TPOAb, thyroid peroxidase antibodies; DIO1, Type 1 Iodothyronine Deiodinase; DIO2, Type 2 Iodothyronine Deiodinase.
