# Supplementary material for: Mendelian randomization study of thyroid function and anti-Müllerian hormone levels
Source: Front Endocrinol (Lausanne). 2023 Jul 20;14:1188284. doi: 10.3389/fendo.2023.1188284 (PMC10400324; doi:10.3389/fendo.2023.1188284)
Supplement: Supplementary file 4 [file Table_3.docx]

**Table S3.** Sensitivity analysis, heterogeneity, and pleiotropy, investigating MR assumption violation

| **Exposure** | **Heterogeneity tests** | | | | | | **Test for directional horizontal pleiotropy** | | | | | | |
| --- | --- | --- | --- | --- | --- | --- | --- | --- | --- | --- | --- | --- | --- |
|  | **Inverse variance weighted** | | | **MR Egger** | | | **Egger_**  **intercept** | **se** | **pval** | **MR-PRESSO global** | **MR-PRESSO distortion test** | **MR-PRESSO**  **Outlier test** | |
|  | **Q** | **Q_df** | **Q_ *P*** | **Q** | **Q_df** | **Q_ *P*** |  |  |  | ***P*** | ***P*** | ***P*** | **Outlier SNPs** |
| Normal range TSH,  ThyroidOmics | 56.769 | 53 | 0.336 | 54.205 | 52 | 0.390 | 0.009 | 0.006 | 0.123 | 0.368 | NA | NA | NA |
| AITD | 14.024 | 14 | 0.448 | 13.434 | 13 | 0.415 | 0.009 | 0.013 | 0.463 | 0.443 | NA | NA | NA |
| no-AITD | 40.951 | 38 | 0.342 | 38.845 | 37 | 0.387 | 0.009 | 0.006 | 0.165 | 0.379 | NA | NA | NA |
| Normal range fT4,  ThyroidOmics | 17.299 | 22 | 0.747 | 17.283 | 21 | 0.694 | -0.001 | 0.008 | 0.899 | 0.744 | NA | NA | NA |
| *DIO1+DIO2* | 1.209 | 4 | 0.877 | 1.177 | 3 | 0.759 | 0.003 | 0.018 | 0.870 | 0.928 | NA | NA | NA |
| *no-DIO1+DIO2* | 15.307 | 17 | 0.573 | 15.276 | 16 | 0.505 | 0.002 | 0.010 | 0.862 | 0.511 | NA | NA | NA |
| Subclinical hypothyroidism, ThyroidOmics | 2.332 | 6 | 0.887 | 2.069 | 5 | 0.840 | 0.013 | 0.024 | 0.630 | 0.869 | NA | NA | NA |
| Subclinical hyperthyroidism, ThyroidOmics | 4.238 | 6 | 0.644 | 3.521 | 5 | 0.620 | -0.023 | 0.027 | 0.436 | 0.700 | NA | NA | NA |
| Overt hypothyroidism,  23andMe | 10.463 | 12 | 0.575 | 10.026 | 11 | 0.528 | 0.006 | 0.010 | 0.522 | 0.649 | NA | NA | NA |
| Normal range TSH,  HUNT | 47.154 | 37 | 0.122 | 45.994 | 36 | 0.123 | 0.006 | 0.007 | 0.347 | 0.136 | NA | NA | NA |
| Full range TSH,  HUNT | 48.412 | 38 | 0.120 | 47.532 | 37 | 0.115 | 0.005 | 0.007 | 0.413 | 0.135 | NA | NA | NA |
| Full range TSH,  HUNT, < 50 years old | 28.837 | 24 | 0.226 | 26.923 | 23 | 0.259 | 0.011 | 0.009 | 0.214 | 0.242 | NA | NA | NA |
| Full range TSH, HUNT+MGI+ThyroidOmics | 97.343 | 80 | 0.091 | 96.987 | 79 | 0.083 | 0.002 | 0.004 | 0.592 | 0.096 | NA | NA | NA |

Abbreviations: MR, mendelian randomization; Q, Cochran’s *Q* statistic; TSH, thyroid stimulating hormone; fT4, Free Thyroxine; HUNT, a longitudinal population health study in Norway; MGI, Michigan Genomics Initiative; TSH, Thyroid-Stimulating Hormone; fT4, Free Thyroxine; AITD, autoimmune thyroid disease; *DIO1*, Type 1 Iodothyronine Deiodinase; *DIO2*, Type 2 Iodothyronine Deiodinase.
