# Supplementary material for: Mendelian randomization study of thyroid function and anti-Müllerian hormone levels
Source: Front Endocrinol (Lausanne). 2023 Jul 20;14:1188284. doi: 10.3389/fendo.2023.1188284 (PMC10400324; doi:10.3389/fendo.2023.1188284)
Supplement: Supplementary file 5 [file Table_4.docx]

**Table S4.** Results of MR-PRESSO analysis between liability to thyroid function and circulating AMH levels.

| **Exposure** | **N SNPs** | **MR-PRESSO** | | |
| --- | --- | --- | --- | --- |
|  |  | **Beta** | **CI** | ***P*** |
| Normal range TSH, ThyroidOmics | 54 | -0.019 | -0.089- 0.0874 | 0.606 |
| AITD | 15 | -0.096 | -0.223- 0.096 | 0.161 |
| no-AITD | 39 | -0.096 | -0.223- 0.031 | 0.161 |
| Normal range fT4, ThyroidOmics | 23 | 0.006 | -0.075- 0.127 | 0.890 |
| *DIO1*+*DIO2* | 5 | -0.018 | -0.103- 0.110 | 0.698 |
| no-*DIO1*+*DIO2* | 18 | -0.106 | -0.220- 0.066 | 0.085 |
| Subclinical hypothyroidism, ThyroidOmics | 7 | -0.023 | -0.061- 0.035 | 0.292 |
| Subclinical hyperthyroidism, ThyroidOmics | 7 | 0.001 | -0.039- 0.060 | 0.969 |
| Overt hypothyroidism, 23andMe | 13 | -0.020 | -0.076- 0.066 | 0.505 |
| Normal range TSH, HUNT | 38 | -0.024 | -0.095- 0.083 | 0.515 |
| Full range TSH, HUNT | 39 | -0.025 | -0.093- 0.078 | 0.476 |
| Full range TSH, HUNT, < 50 years old | 25 | -0.017 | -0.084- 0.084 | 0.621 |
| Full range TSH, HUNT+MGI+ThyroidOmics | 81 | -0.042 | -0.108- 0.058 | 0.218 |

Abbreviations: MR-PRESSO, MR Pleiotropy RESidual Sum and Outlier; TSH, thyroid stimulating hormone; fT4, Free Thyroxine; HUNT, a longitudinal population health study in Norway; MGI, Michigan Genomics Initiative; TSH, Thyroid-Stimulating Hormone; fT4, Free Thyroxine; AITD, autoimmune thyroid disease; *DIO1*, Type 1 Iodothyronine Deiodinase; *DIO2*, Type 2 Iodothyronine Deiodinase.
