# Supplementary figures and images for: Mendelian randomization study of thyroid function and anti-Müllerian hormone levels
Source: Front Endocrinol (Lausanne). 2023 Jul 20;14:1188284. doi: 10.3389/fendo.2023.1188284 (PMC10400324; doi:10.3389/fendo.2023.1188284)

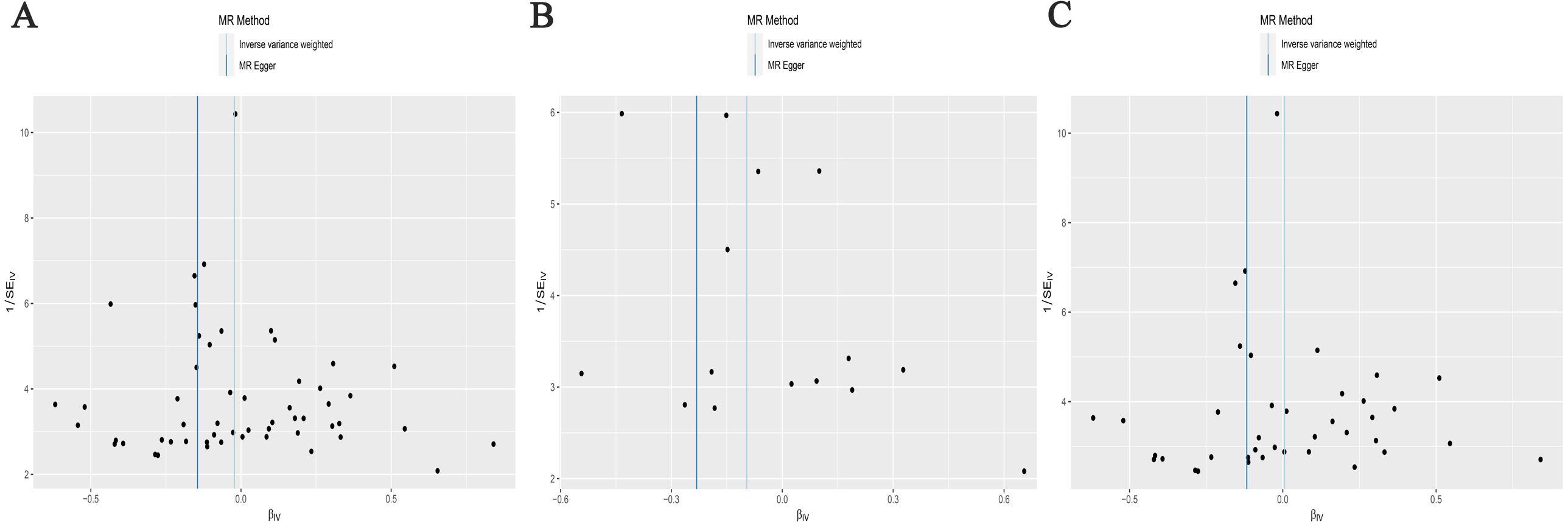

Supplement: Supplementary Figure 1 — Funnel plots of the effect of normal range TSH on AMH levels in ThyroidOmics dataset. (A) Normal range TSH. (B) Normal range TSH with AITD. (C) Normal range TSH without AITD. AMH, anti-Müllerian hormone; TSH, thyroid stimulating hormone; AITD, autoimmune thyroid disease. [file Image_1.tif]

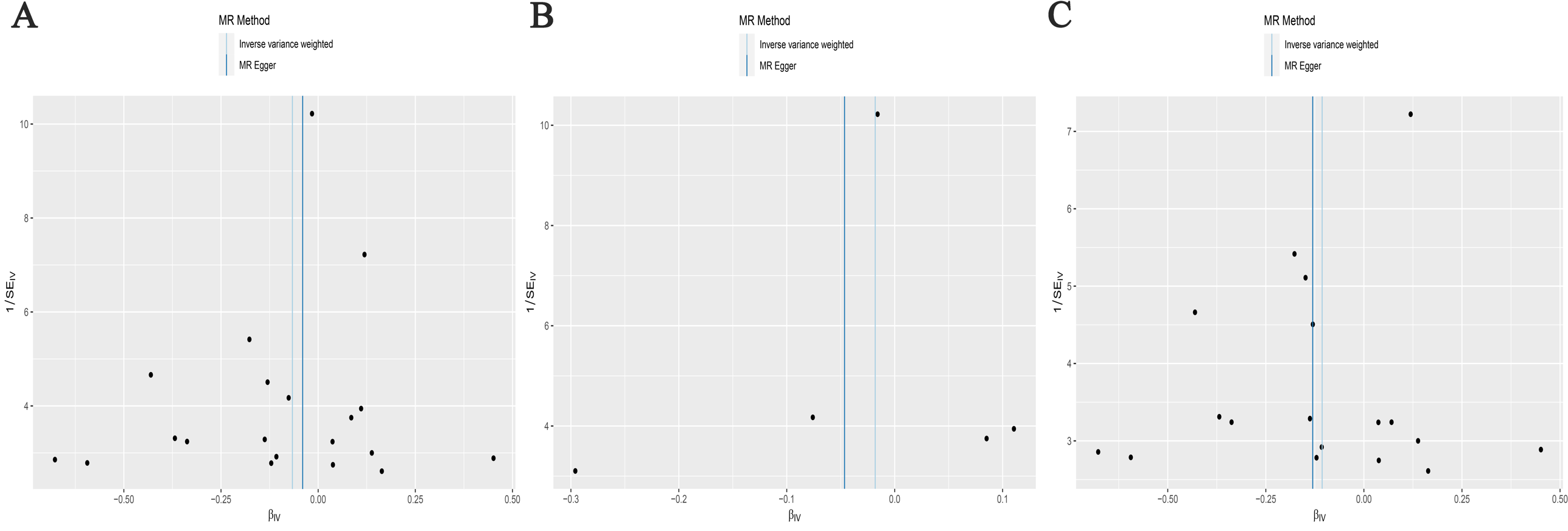

Supplement: Supplementary Figure 2 — Funnel plots of the effect of normal range fT4 on AMH levels in ThyroidOmics dataset. (A) Normal range fT4. (B) Normal range fT4 with DIO1+DIO2. (C) Normal range fT4 without DIO1+DIO2. AMH, anti-Müllerian hormone; fT4, free thyroxine; DIO1, Type 1 Iodothyronine Deiodinase; DIO2, Type 2 Iodothyronine Deiodinase. [file Image_2.tif]

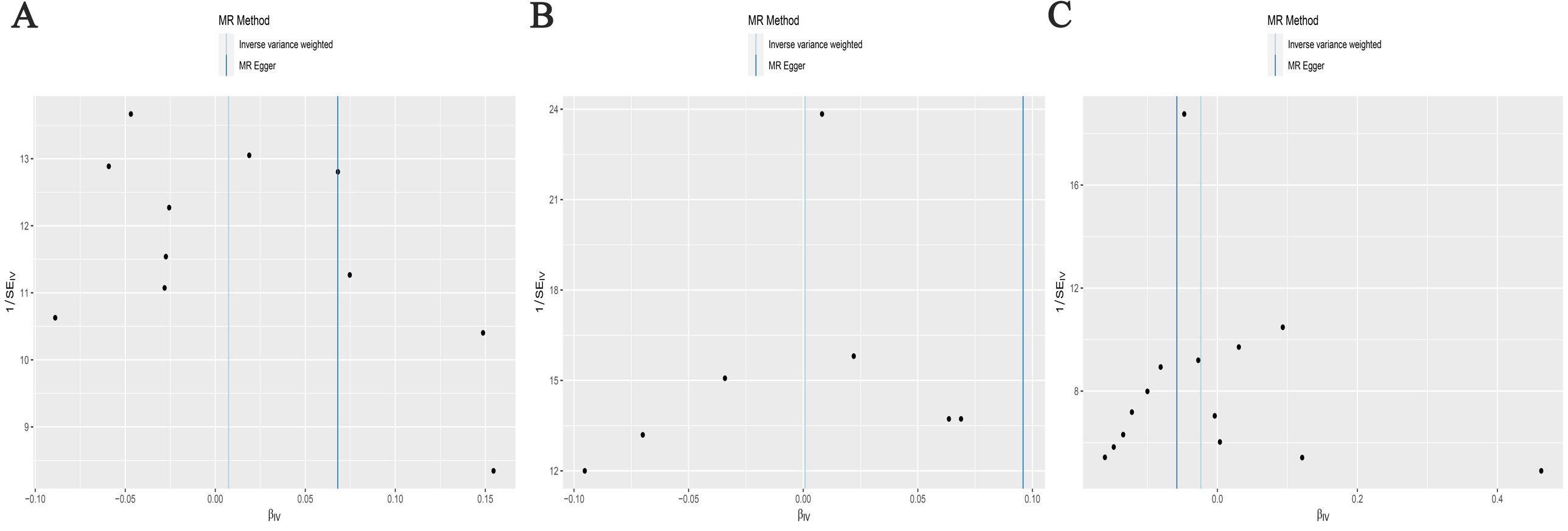

Supplement: Supplementary Figure 3 — Funnel plots of the effect of thyroid function on AMH levels. (A) Subclinical hypothyroidism, ThyroidOmics. (B) Subclinical hyperthyroidism, ThyroidOmics. (C) Overt hypothyroidism, 23andMe. AMH, anti-Müllerian hormone. [file Image_3.tif]

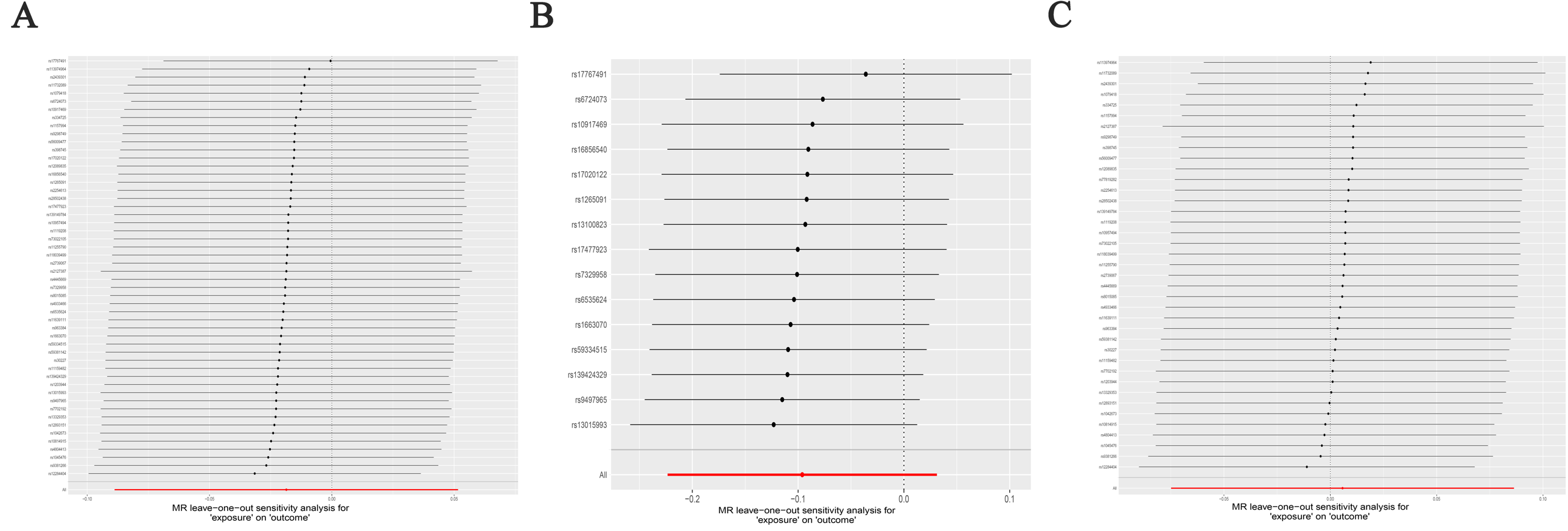

Supplement: Supplementary Figure 4 — Funnel plots of the effect of thyroid function on AMH levels in the subgroup analyses for TSH. A Normal range TSH, HUNT. B Full range TSH, HUNT. C Full range TSH, HUNT, < 50 years old. D Full range TSH, HUNT+MGI+ThyroidOmics. TSH, thyroid stimulating hormone; HUNT, a longitudinal population health study in Norway; MGI, Michigan Genomics Initiative. [file Image_4.tif]

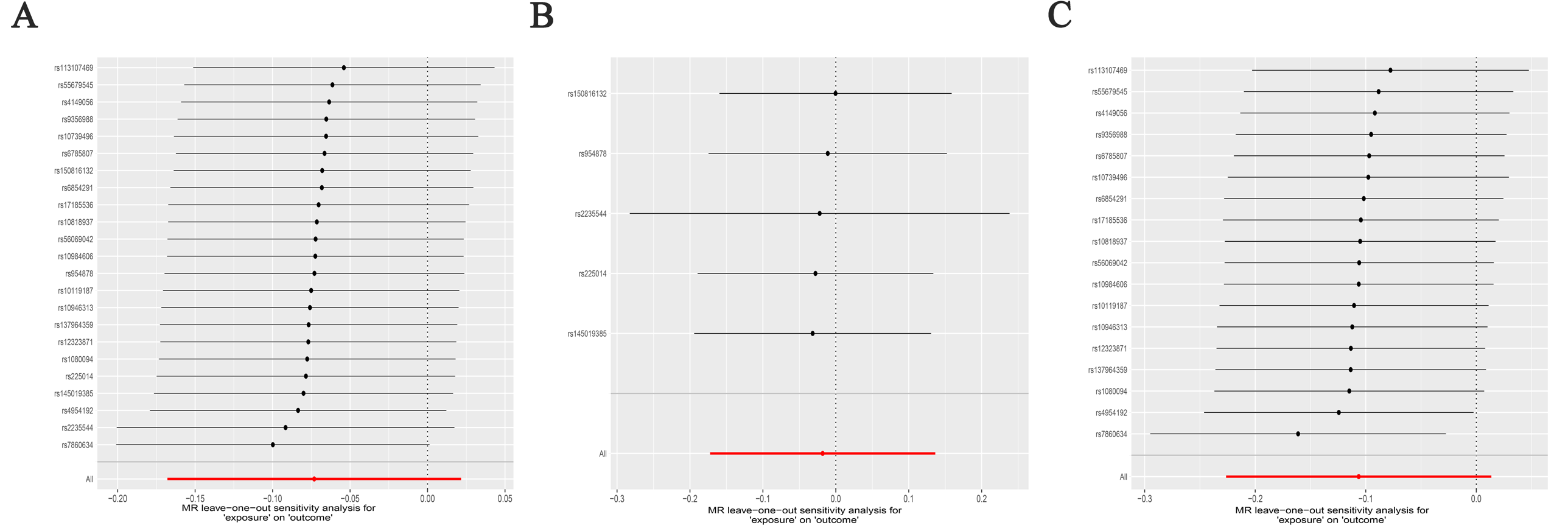

Supplement: Supplementary Figure 5 — Leave-one-out plots of the effect of normal range TSH on AMH levels in ThyroidOmics dataset. (A) Normal range TSH. (B) Normal range TSH with AITD. (C) Normal range TSH without AITD. AMH, anti-Müllerian hormone; TSH, thyroid stimulating hormone; AITD, autoimmune thyroid disease. [file Image_5.tif]

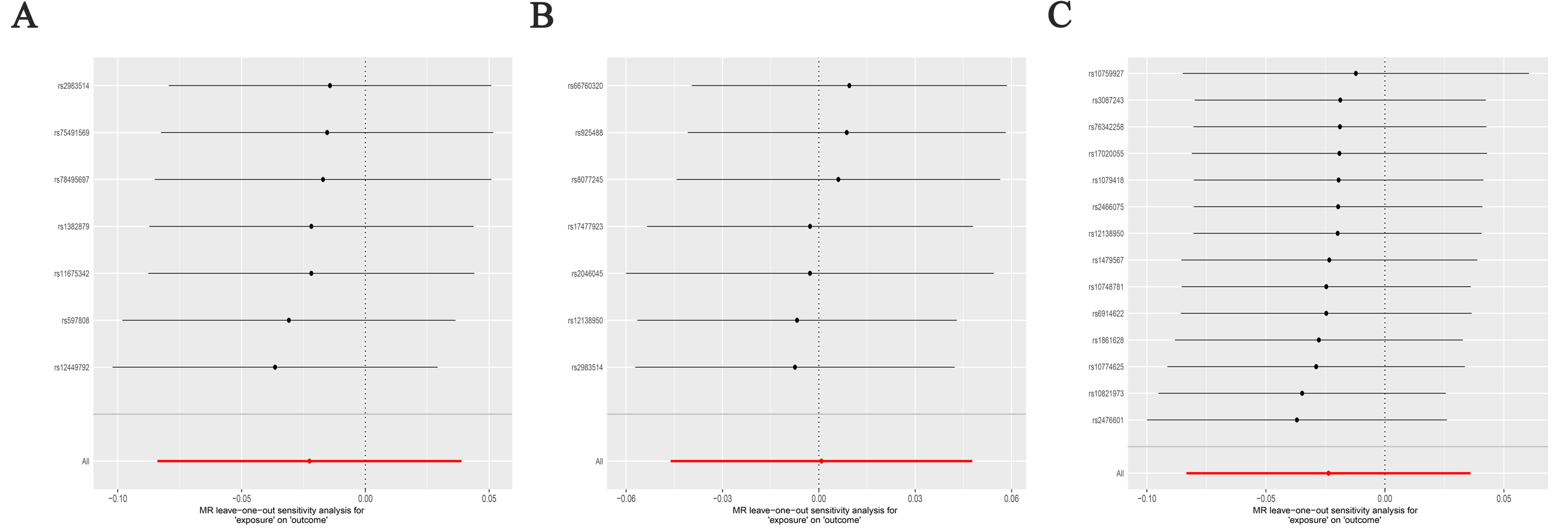

Supplement: Supplementary Figure 6 — Leave-one-out plots of the effect of normal range fT4 on AMH levels in ThyroidOmics dataset. (A) Normal range fT4. (B) Normal range fT4 with DIO1+DIO2. (C) Normal range fT4 without DIO1+DIO2. AMH, anti-Müllerian hormone; fT4, free thyroxine; DIO1, Type 1 Iodothyronine Deiodinase; DIO2, Type 2 Iodothyronine Deiodinase. [file Image_6.tif]

A

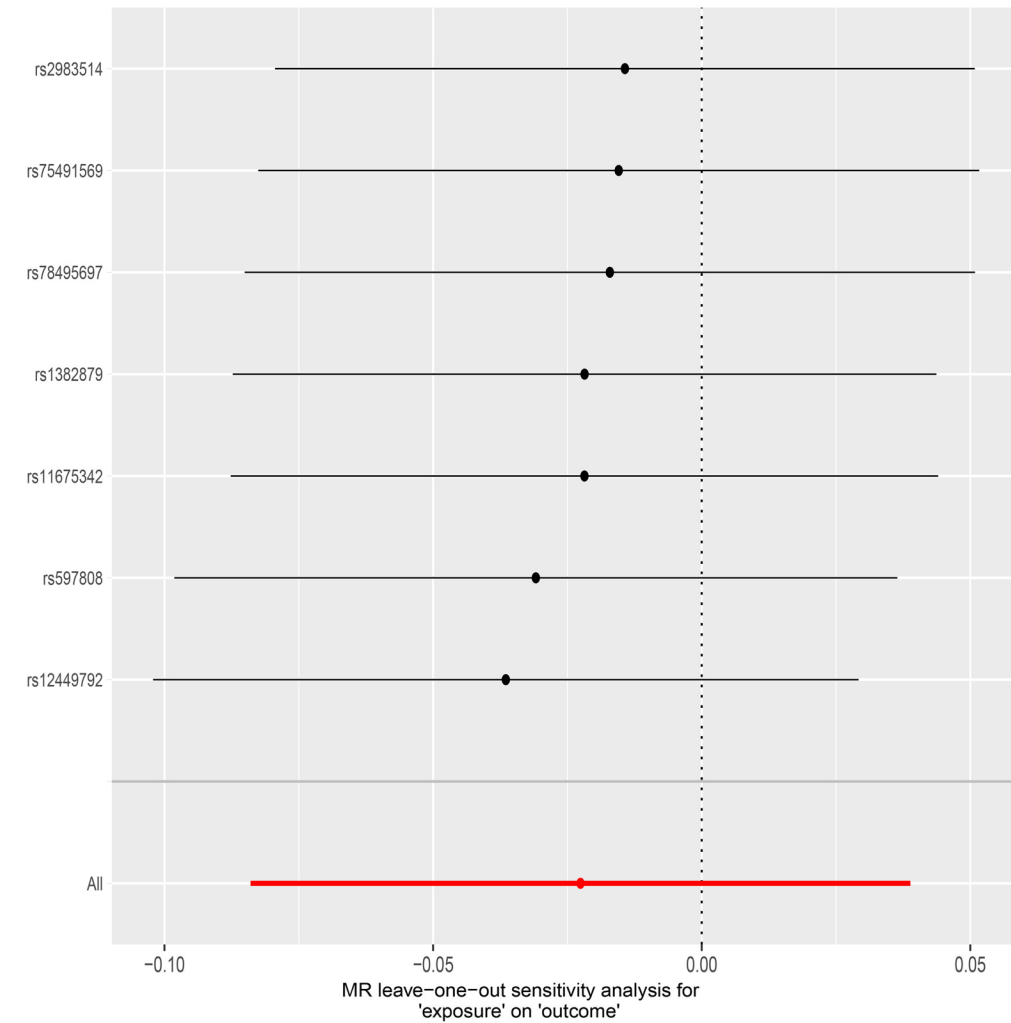

B

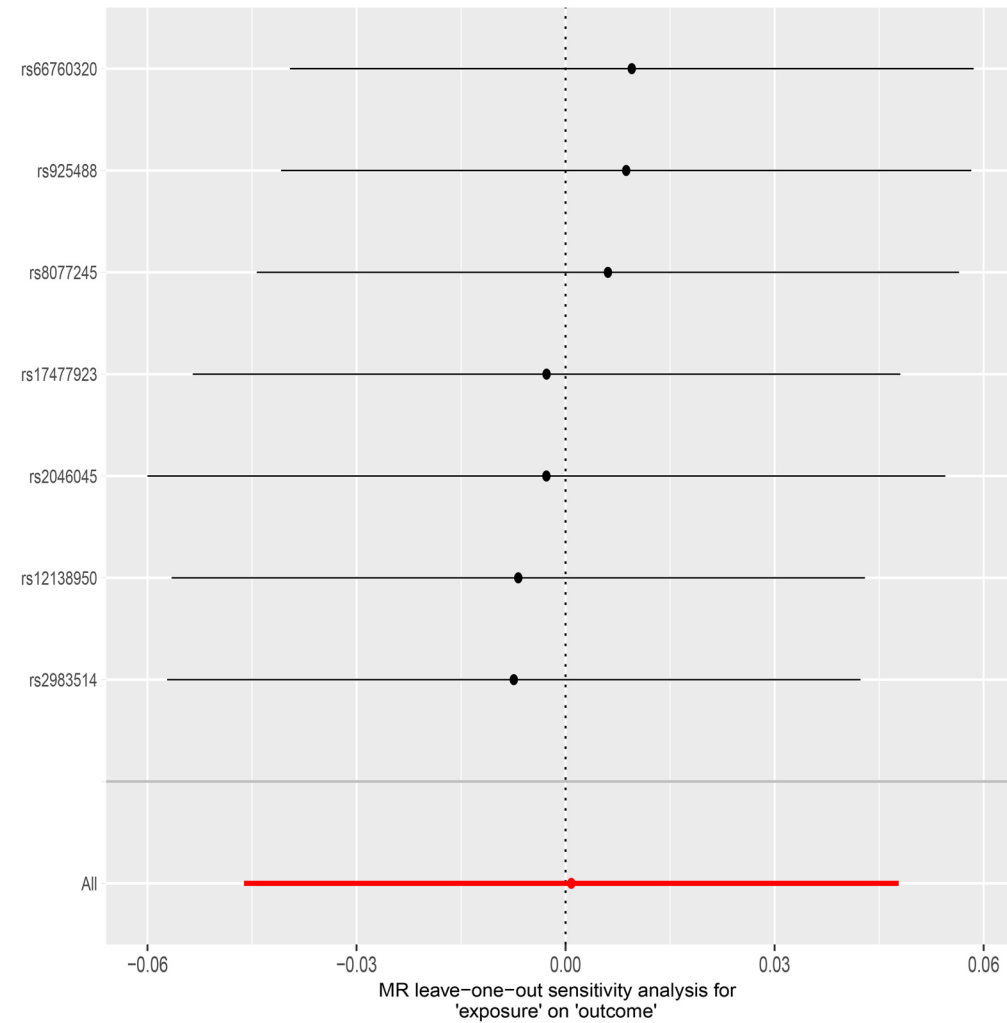

C

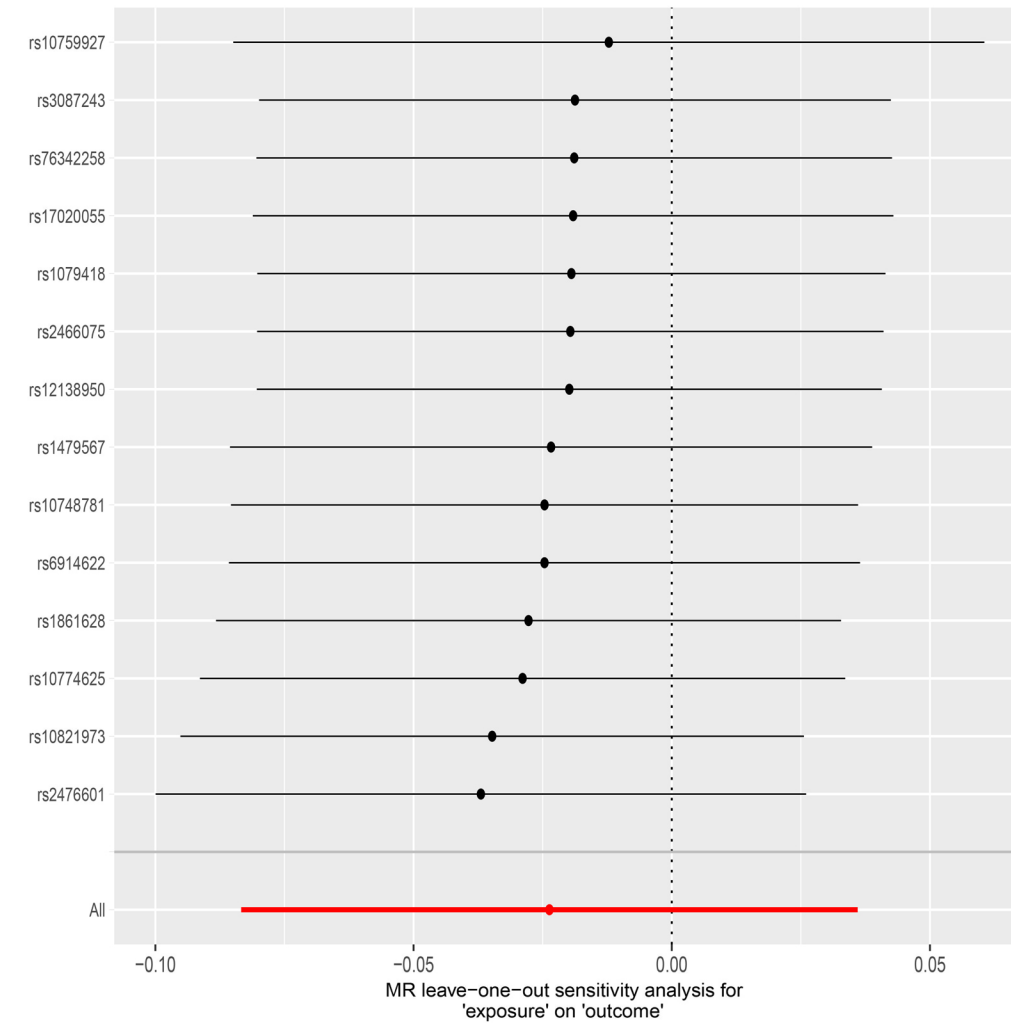

Supplement: Supplementary Figure 7 — Leave-one-out plots of the effect of thyroid function on AMH levels. (A) Subclinical hypothyroidism, ThyroidOmics. (B) Subclinical hyperthyroidism, ThyroidOmics. (C) Overt hypothyroidism, 23andMe. AMH, anti-Müllerian hormone. [file Image_7.pdf]

A

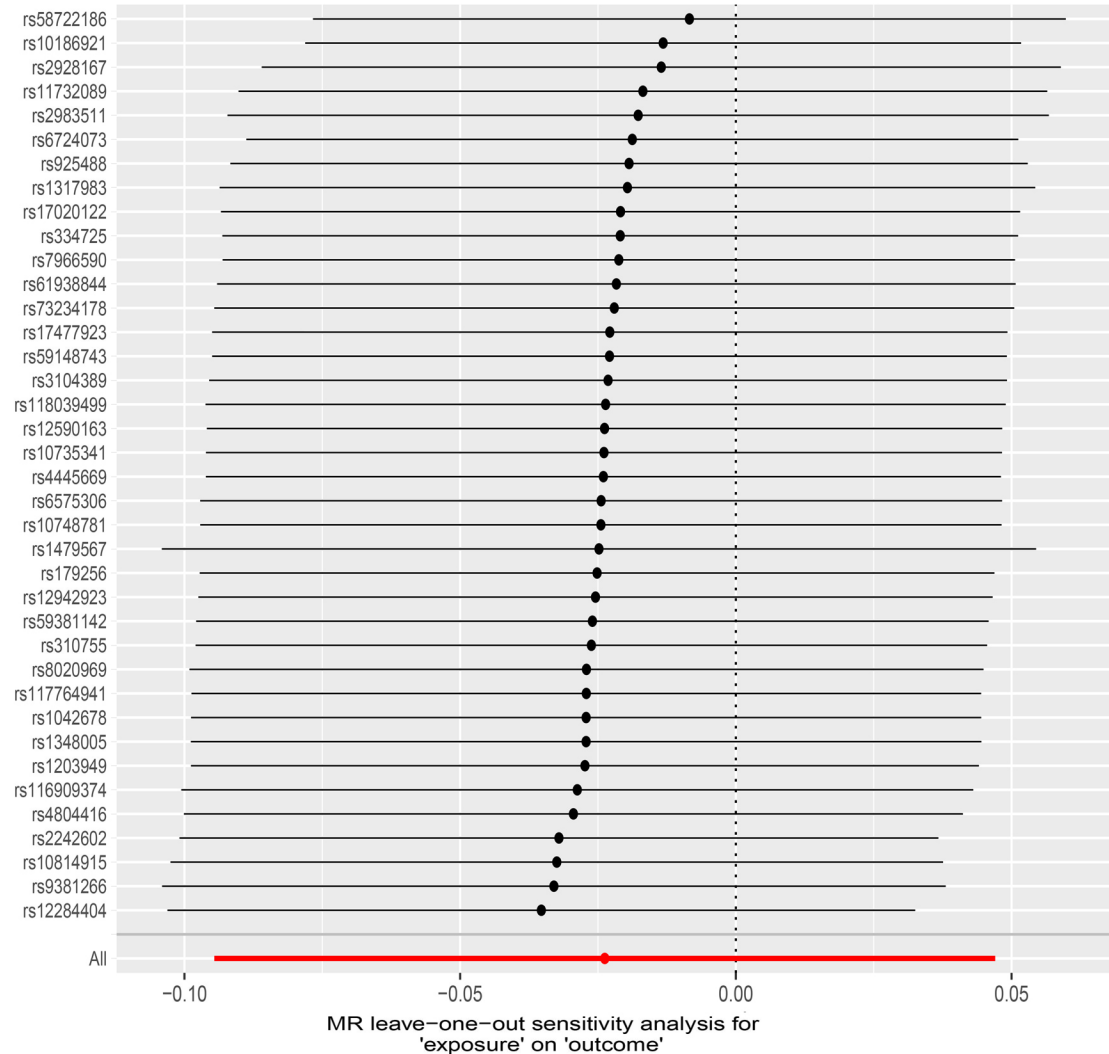

B

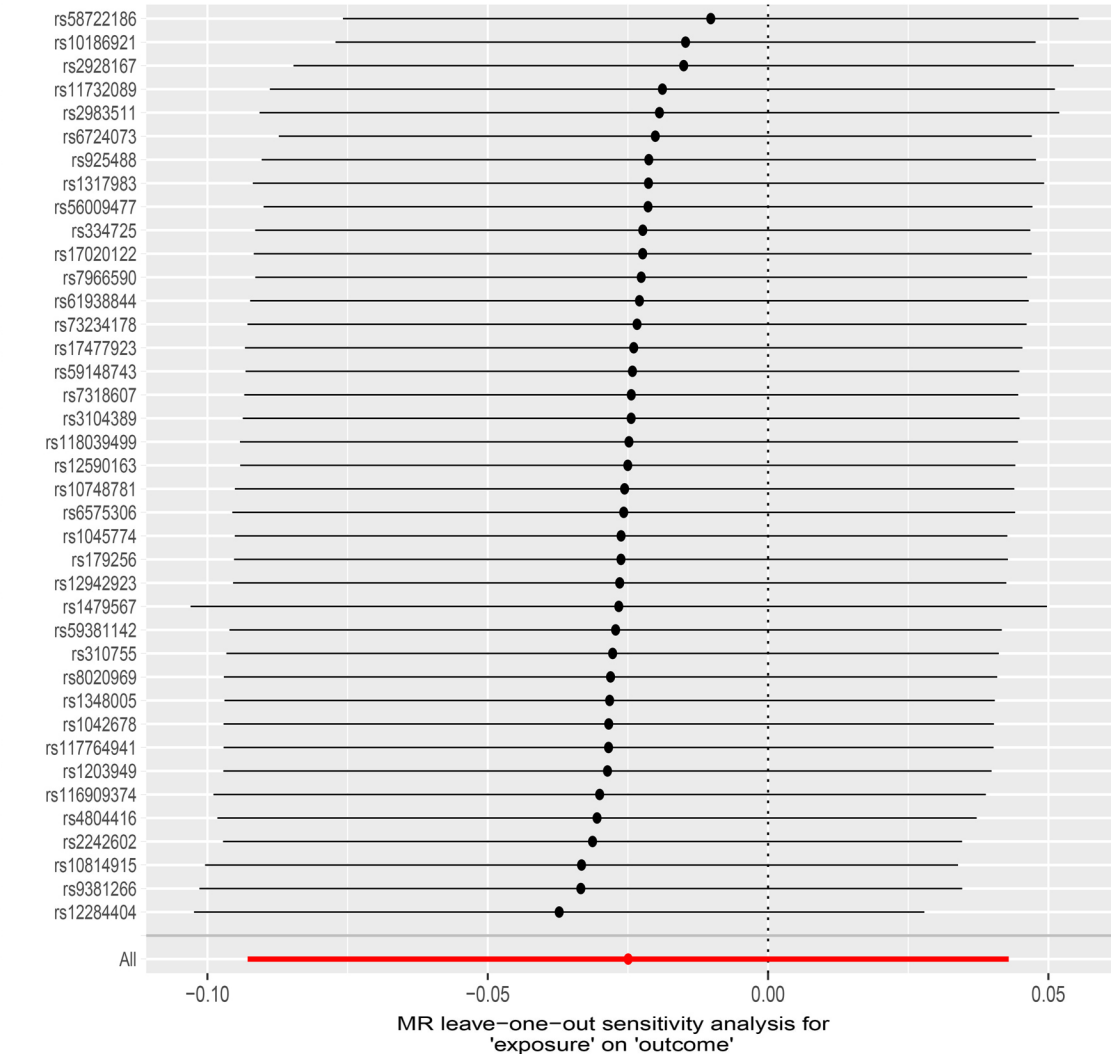

C

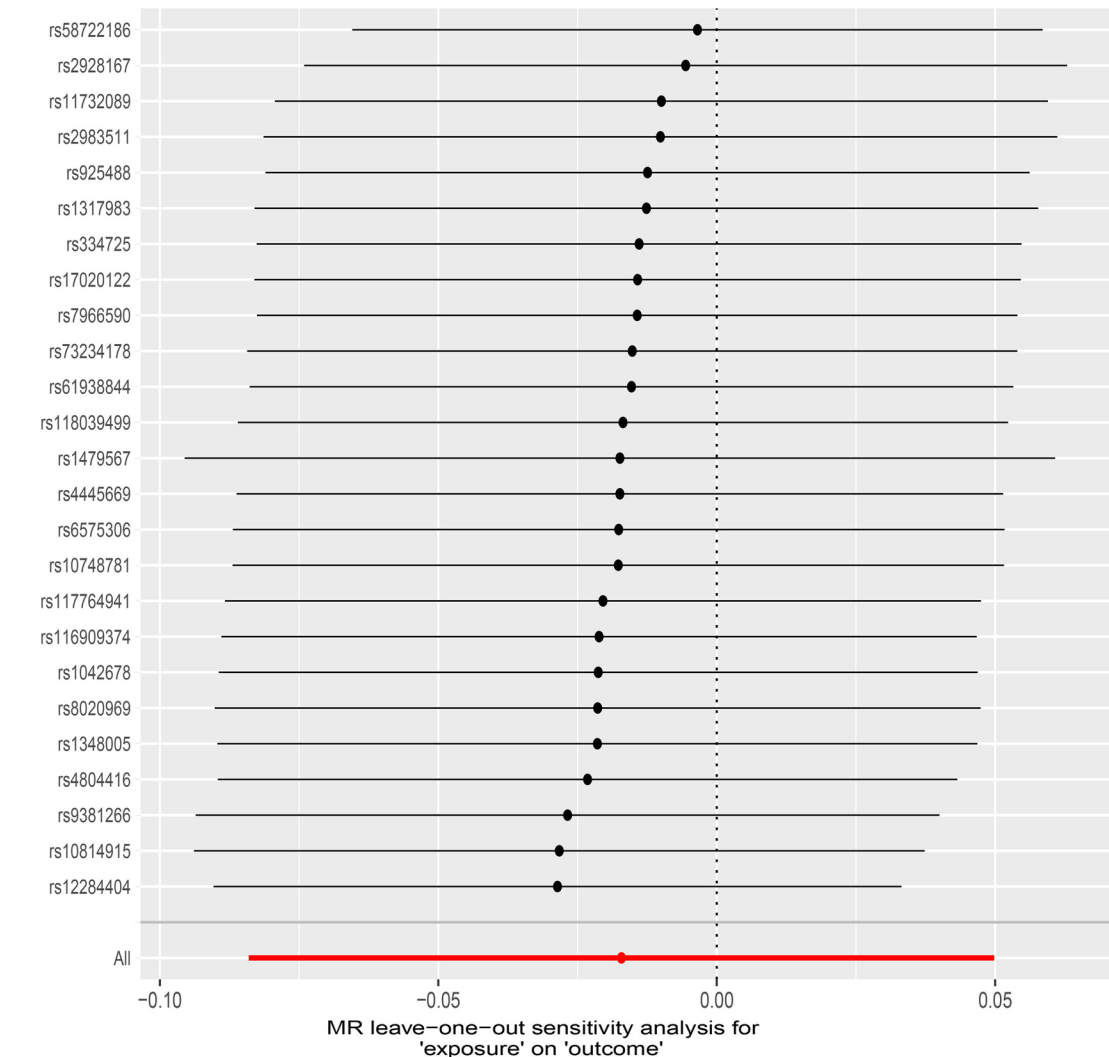

D

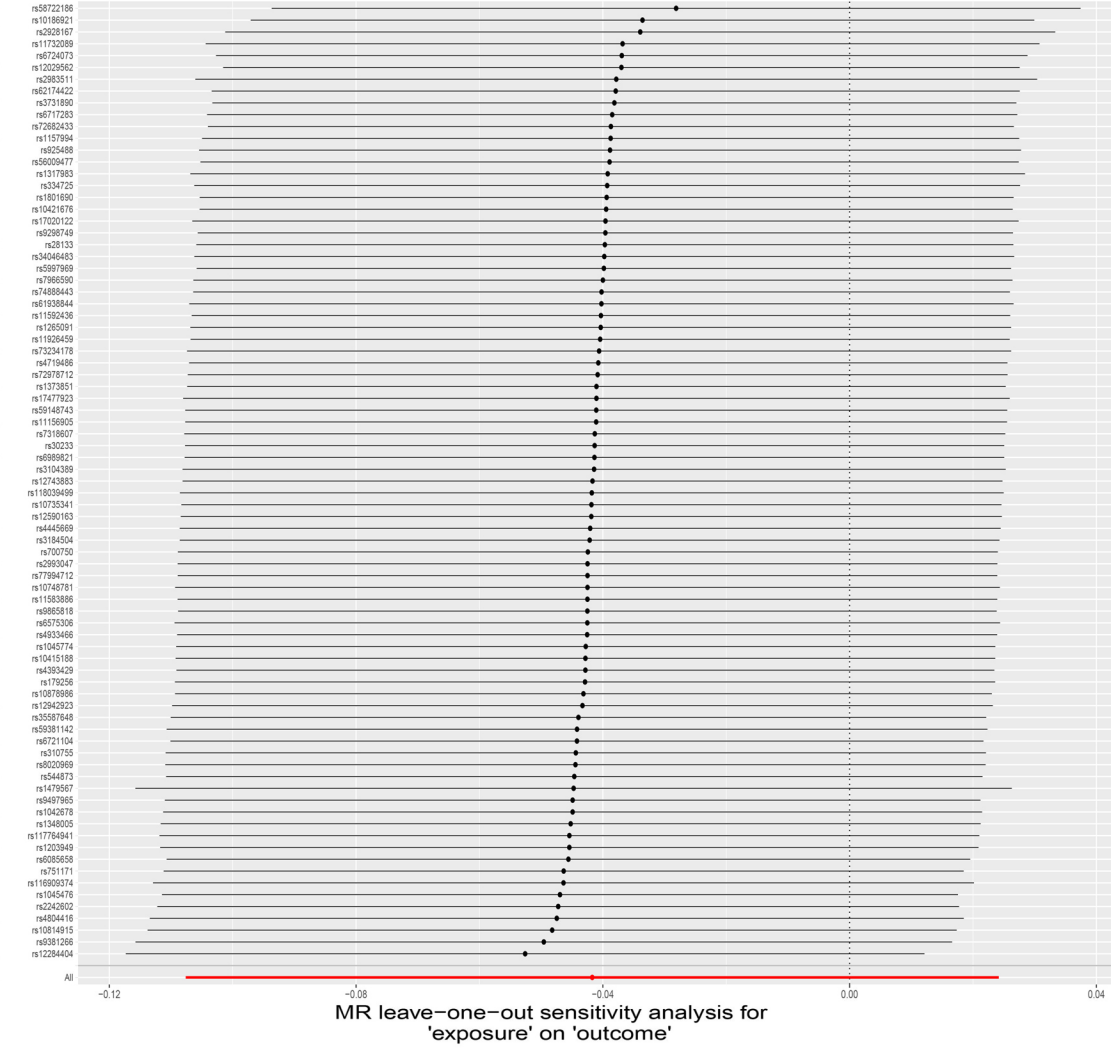

Supplement: Supplementary Figure 8 — Leave-one-out plots of the effect of thyroid function on AMH levels in the subgroup analyses for TSH. (A) Normal range TSH, HUNT. (B) Full range TSH, HUNT. (C) Full range TSH, HUNT, < 50 years old. (D) Full range TSH, HUNT+MGI+ThyroidOmics. [file Image_8.pdf]
